# Particle characterization and toxicity in C57BL/6 mice following instillation of five different diesel exhaust particles designed to differ in physicochemical properties

**DOI:** 10.1186/s12989-020-00369-9

**Published:** 2020-08-08

**Authors:** Katja Maria Bendtsen, Louise Gren, Vilhelm Berg Malmborg, Pravesh Chandra Shukla, Martin Tunér, Yona J. Essig, Annette M. Krais, Per Axel Clausen, Trine Berthing, Katrin Loeschner, Nicklas Raun Jacobsen, Henrik Wolff, Joakim Pagels, Ulla Birgitte Vogel

**Affiliations:** 1 National Research Centre for the Working Environment, Lersø Parkallé 105, DK-2100 Copenhagen, Denmark; 2 Division of Ergonomics and Aerosol Technology, Lund University, Box 117, 221 00 Lund, Sweden; 3 NanoLund, Lund University, Box 118, 221 00 Lund, Sweden; 4 Division of Combustion Engines, Lund University, Box 117, 221 00 Lund, Sweden; 5 Department for Occupational and Environmental Medicine, Institute of Laboratory Medicine, Lund University, 221 00 Lund, Sweden; 6 Research Group for Nano-Bio Science, National Food Institute,Technical University of Denmark, Kemitorvet, Building 201, DK-2800 Kgs. Lyngby, Denmark; 7 Finnish Institute of Occupational Health, Työterveyslaitos, P.O. Box 40, FI-00032 Helsinki, Finland; 8 Department of Health Technology, Technical University of Denmark, DK-2800 Kgs. Lyngby, Denmark

**Keywords:** Diesel exhaust particles - ultrafine particles, Toxicity, Intratracheal instillation, Exhaust gas recirculation, Renewable diesel fuels

## Abstract

**Background:**

Diesel exhaust is carcinogenic and exposure to diesel particles cause health effects. We investigated the toxicity of diesel exhaust particles designed to have varying physicochemical properties in order to attribute health effects to specific particle characteristics. Particles from three fuel types were compared at 13% engine intake O_2_ concentration: MK1 ultra low sulfur diesel (DEP13) and the two renewable diesel fuels hydrotreated vegetable oil (HVO13) and rapeseed methyl ester (RME13). Additionally, diesel particles from MK1 ultra low sulfur diesel were generated at 9.7% (DEP9.7) and 17% (DEP17) intake O_2_ concentration. We evaluated physicochemical properties and histopathological, inflammatory and genotoxic responses on day 1, 28, and 90 after single intratracheal instillation in mice compared to reference diesel particles and carbon black.

**Results:**

Moderate variations were seen in physical properties for the five particles: primary particle diameter: 15–22 nm, specific surface area: 152–222 m^2^/g, and count median mobility diameter: 55–103 nm. Larger differences were found in chemical composition: organic carbon/total carbon ratio (0.12–0.60), polycyclic aromatic hydrocarbon content (1–27 μg/mg) and acid-extractable metal content (0.9–16 μg/mg). Intratracheal exposure to all five particles induced similar toxicological responses, with different potency. Lung particle retention was observed in DEP13 and HVO13 exposed mice on day 28 post-exposure, with less retention for the other fuel types. RME exposure induced limited response whereas the remaining particles induced dose-dependent inflammation and acute phase response on day 1. DEP13 induced acute phase response on day 28 and inflammation on day 90. DNA strand break levels were not increased as compared to vehicle, but were increased in lung and liver compared to blank filter extraction control. Neutrophil influx on day 1 correlated best with estimated deposited surface area, but also with elemental carbon, organic carbon and PAHs. DNA strand break levels in lung on day 28 and in liver on day 90 correlated with acellular particle-induced ROS.

**Conclusions:**

We studied diesel exhaust particles designed to differ in physicochemical properties. Our study highlights specific surface area, elemental carbon content, PAHs and ROS-generating potential as physicochemical predictors of diesel particle toxicity.

## Introduction

The classification of diesel exhaust as carcinogenic [1] and the reported diesel particle airway toxicity and systemic effects in both humans [2–4] and in mice [5–12] necessitates further studies. The ultrafine particle fraction has been suggested to be a main mediator of the carcinogenic effects and to contribute to other adverse health effects [13], but it is less known if it can be related to specific physicochemical particle properties.

Diesel engine exhaust consists of a particulate phase containing insoluble high surface area carbonaceous particles (elemental carbon; EC, also known as black carbon) with absorbed metal oxides and an adsorbed liquid fraction containing low volatility organic matter, including polycyclic aromatic hydrocarbons (PAH) formed in the combustion process and branched alkanes originating from the lubrication oil. In addition, diesel exhaust consists of gases including carbon monoxide, nitrogen oxides (NOx), and volatile organic compounds (VOCs) [14, 15]. Particulate matter and gas emissions can be reduced either by altering the combustion process or by installing after-treatment systems. Exhaust gas recirculation (EGR) is commonly used as a NOx reduction technique, but as the reduction of oxygen concentration and temperature reduce NO_x_ emissions, soot emissions increase [13, 16]. Hence, the physicochemical characteristics of the emitted particles, such as content of PAH, metals and ratio of elemental and organic carbon [14, 17, 18], depend on engine combustion conditions. Particle size and thereby specific surface area (SSA) is a driver of pulmonary inflammation [19] and acute phase response [20, 21] whereas certain metals and PAH, as well as ROS formation are linked to genotoxicity [16, 22, 23]. Several PAH compounds including benzo[a]pyrene are classified as carcinogenic or possibly carcinogenic by IARC [24].

Emissions also depend on fuel type [18, 25–27], and recently, renewable diesel fuels have been introduced on large scales to replace fossil diesel [28–30]. There is incomplete knowledge on the potential adverse health effects of emissions from these new types of renewable diesel fuels, especially regarding gentoxicity and carcinogenicity [23]. Renewable diesel fuels are based on vegetable oils, animal fats or waste products and are used in conventional engines as full substitution or in blends. Rapeseed methyl ester is a fatty acid methyl ester and differs from fossil diesel, as it has a high O_2_ content in the fuel (~ 10%). More recently, second generation renewable diesel fuels have become available where the oxygen content is removed by hydrogen treatment. One such example is hydrogen treated vegetable oil (HVO) which is a synthetic/paraffinic diesel produced from plant and animal sources and chemically similar to fossil diesel, except it has no aromatic content and shorter carbon chains [26].

In this study, we investigated the toxicological effects following pulmonary exposure to diesel exhaust particles collected without after-treatment from a controlled modern heavy-duty diesel engine in a laboratory environment. The particle production and collection was described in detail previously [31]. Briefly, the engine was operated in different modes to vary the physical and chemical properties of five different diesel exhaust particles (DEP). Three different levels of EGR were chosen in order to generate diesel exhaust particles with a) high fraction of PAH and refractory organic carbon (OC) relative to EC, b) high fraction of EC and c) high fractions of lubrication oil related OC and metals relative to EC. The three EGR levels corresponded to engine intake O_2_ concentrations of 9.7% (DEP9.7), 13% (DEP13) and 17% (DEP17). For these EGR levels, the engine was fueled with petroleum-based ultralow-sulfur diesel of Swedish MK1 standard. In order to investigate the effect of renewable fuels, two additional particle types were generated by operating the engine on renewable rapeseed methyl ester (RME13) and hydrotreated vegetable/animal oil (HVO13) at an engine intake O_2_ concentration of 13%. Thus, the diesel exhaust particles were designed to differ in primary particle size and in content of PAHs, OC, EC and metals.

The aims of the study were: 1) to study the toxicity of five diesel exhaust particle samples designed to differ in physicochemical properties and 2) to identify physicochemical properties driving the toxicity of the particles. Toxicity was evaluated in terms of reactive oxygen species generation and inflammatory and genotoxic responses in mice on day 1, 28 and 90 after exposure to 6, 18 and 54 μg collected particles by single intratracheal instillation.

## Results

### Physicochemical properties

The characterization of the five different combustion particles in terms of particle mobility size in air, morphology by transmission electron microscopy (TEM), elemental carbon (EC)/organic carbon (OC) to total carbon (TC) ratio, PAH and metal contents are shown in Table 1 as previously reported [31]. Overall, combustion conditions were the most important determinant for all particle characteristics. The combustion conditions heavily affected the mobility size, OC, EC, metal and PAH contents. The engine emissions measured as total PM1 were reduced by 65% for the renewable diesel fuels compared to the fossil diesel fuel (Table 1).

**Table 1:** Mass, size, carbon composition and surface area of particles

Particles	DEP			HVO	RME	*Ref. Carbon black Printex90*
Fuel	MK1 low sulfur diesel			Hydrotreated vegetable oil	Rapeseed methyl ester	–
Intake O_2_ (%)	**9.7**	**13**	**17**	**13**	**13**	**–**
Average emitted exhaust PM1 mass concentration (mg/m^3^)^a^	23	96	3	34	34	–
Particle mobility diameter (GMD) (nm)^b^	55 ± 9	104 ± 7	62 ± 4	90 ± 5	70 ± 3	–
Primary particle diameter (GMD_p_) (nm) ^c^	22 [21,23]	17 [16,19]	16 [15,17]	21 [19, 23]	15 [14, 16]	15 [14, 15]
Estimated specific surface area (SSA) (m^2^/g)^c^	152 [143, 161]	191 [177, 206]	207 [191, 224]	160 [146, 174]	222 [203, 243]	230 [217, 243]
Elemental to total carbon (EC/TC)	0.35	0.88	0.60	0.72	0.68	
Organic to total carbon (OC/TC)	0.65	0.12	0.40	0.28	0.32	

^a^The average exhaust PM mass concentration (mg/m^3^), before dilution and particle mobility diameter (nm) with ±1 std. dev. in the time series. The GMD of the soot primary particle size was estimated from the TEM images and the specific surface area (SSA) was estimated from the primary particle size distributions. For the primary particle diameter and SSA, intervals in brackets represent the 95% confidence interval of the distribution parameters in the lognormal fitting procedure. The elemental carbon (EC) and organic carbon (OC) to total carbon (TC) fractions are measured in the extracted PM

^b^Mobility particle (agglomerate) size based on the number concentration. Measured with the DMS in the dilution tunnel when the particles were airborne. ^c^Geometric mean diameter. Measured on collected samples without the extraction process

Analysis of OC, EC, metal- and PAH content was carried out on extracted particles, whereas TEM and mobility size distribution analyses was done on diluted exhaust particles.

#### Electron microscopy

**Fig. 1:**
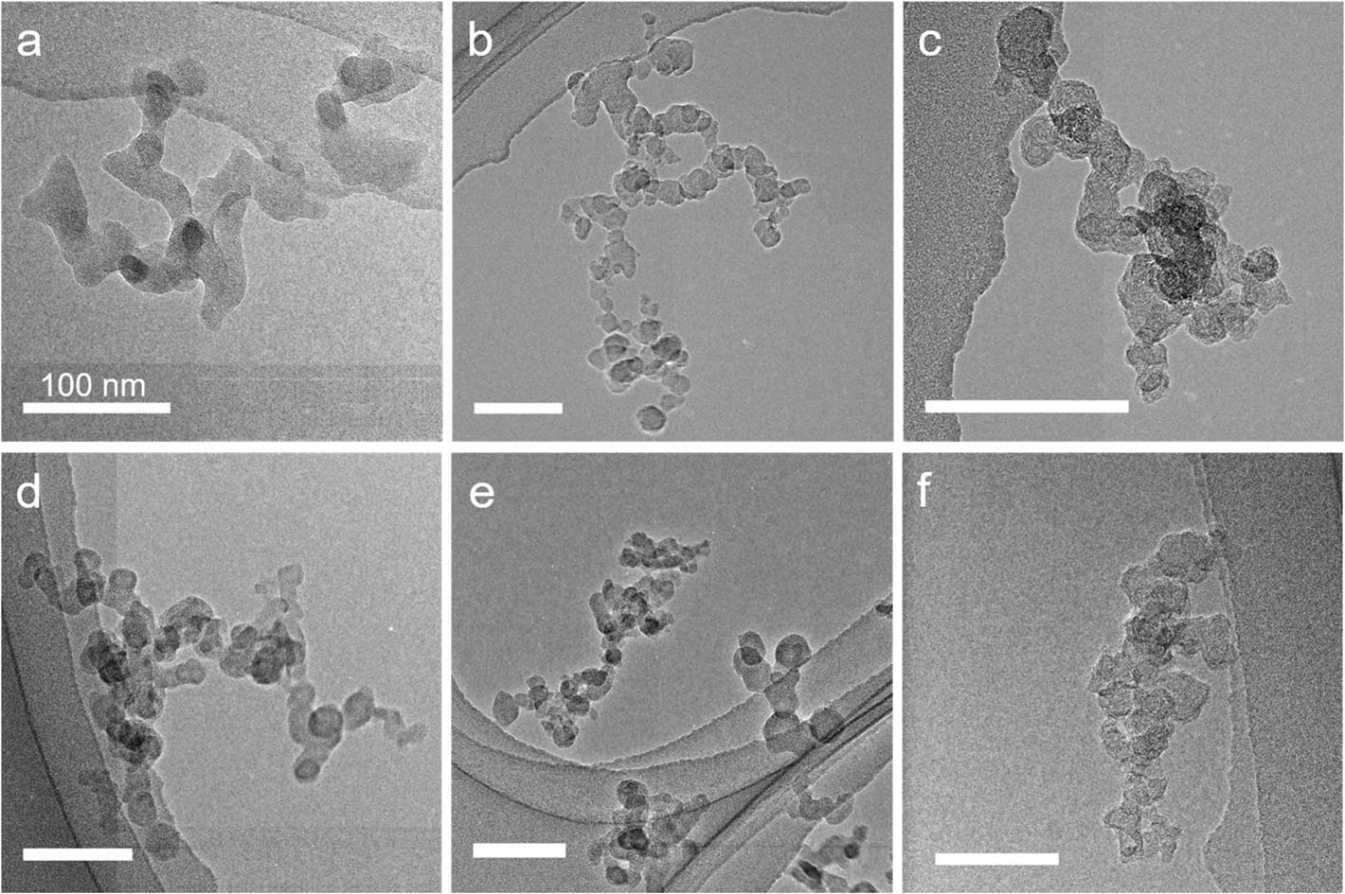
TEM images of DEP9.7 (**a**), DEP13 (**b**), and DEP17 (**c**), HVO13 (**d**), RME13 (**e**) and CB (**f**). The morphology of the particles generated with 13 and 17% O_2_ conditions (**b**, **c**, **d**, and **e**) are similar, while the soot agglomerates generated with 9.7% O_2_ (a) have less defined primary particles and appear more aggregated compared to the other samples

The five different particle samples from experimental combustion emissions and the CB reference sample were visualized by transmission electron microscopy (Fig. 1). The morphology of the particles generated with 13% intake O_2_ concentration, namely DEP13, HVO13 and RME13 (Fig. 1 b, d, e) and DEP17 with 17% intake O_2_ concentration (Fig. 1 c) were all similar in appearance and showed typical soot agglomerates (diameter ~ 50–300 nm) of smaller primary particles (diameter ~ 10–30 nm). In contrast, the soot agglomerates from low temperature combustion (DEP9.7) had less defined primary particles and appeared more fused (bridging between primary particles) compared to the other samples (Fig. 1 a).

#### Organic and elemental carbon, primary particle size and specific surface area

Primary particle size and specific surface area (SSA) were estimated and calculated by measuring the diameters of well-defined spherical primary particles from the TEM images (assuming no voids inside the primary particles). The primary particle size decreased with the intake O_2_ concentration for the MK1 diesel samples. The largest primary particle size was found for HVO13, 23 nm, followed by 17 nm for DEP13 and 16 nm for RME13 (Table 1). Estimated SSA was overall rather similar, with the largest SSA for RME13 with 222 m^2^/g and the lowest for DEP9.7 with 152 m^2^/g. The EC fraction was highest for the particles generated at 13% intake O_2_, and the lowest for 9.7% intake O_2_. The EC fraction was higher for DEP13 compared to HVO13 and RME13.

The specific surface area (SSA) was estimated by using the primary particle size (d_pp) distribution and diesel soot density (ρ_pp) of 1.77 μg/m^3^ [32] with the formula SSA =6/(ρ_pp · d_pp). Data from Gren et al. [31].

#### Metal contents

Semi-quantitative analysis of elemental contents by inductive coupled plasma mass spectrometry (ICP-MS) showed the highest mass fractions for Cu and Fe (Table 2). For Cu, Fe and several other trace elements, DEP17 showed 5–17 fold higher metal mass fractions compared to the other four samples. The emitted exhaust metal mass concentrations (μg/m^3^) were within a factor 2 for all operation points, however the PM1 mass emissions varied strongly (Table 1) and hence the metal mass fraction will be higher for the low mass emitting operation point (DEP17). DEP13 had the lowest mass fraction of Cu. RME13 and DEP13 had the lowest mass fraction of Fe.

**Table 2:** Extracted elemental mass fractions (μg/g)

Particles	DEP			HVO	RME	*Reference values*			
Fuel	MK1 low sulfur diesel			Hydrotreated vegetable oil	Rapeseed methyl ester	*NIST2975*^*a*^	*CB*^*a*^	*Ref.*^*b*^*NIST2975*	*Ref.*^*c*^*CB*
Intake O_2_%	9.7	13	17	13	13				
**V**	14	6	ND	3	2	ND	ND	–	–
**Cr**	8	7	52	11	7	7/4	ND	–	–
**Mn**	92	53	ND	39	43	6/3	1/0	–	–
**Fe**	220	137	2115	247	116	663/516	9/12	0.0 ± 0.0	< 1
**Co**	2	1	88	1	1	0/0	ND	–	< 1
**Ni**	15	6	118	9	25	4/4	ND/1	–	–
**Cu**	2.349	629	13,160	1632	2291	23/13	10/1	0.0 ± 13	11
**Ga**	1	1	1	1	1	ND	ND/0	0.1 ± 0.1	< 1
**As**	ND	ND	ND	ND	ND	ND	1/2	0.5 ± 0.7	< 2
**Se**	2	0	ND	ND	0	ND	ND	0.9 ± 0.6	< 1
**Rb**	2	1	1	1	1	13,926/17,003	ND	16 ± 4	< 2
**Sr**	99	54	ND	41	37	2/1	1/1	–	–
**Ag**	0	0	1	0	0	0/0	0/0	–	< 2
**Cd**	ND	ND	ND	ND	ND	ND	ND/0	–	< 10
**In**	0	0	0	0	0	0/0	0/0	–	–
**Cs**	0	0	ND	0	0	ND	ND	–	–
**Ba**	15	10	ND	9	6	26/ND	ND	–	–
**Hg**	0	0	ND	0	ND	0/0	0/0	–	< 0.4
**Tl**	0	0	0	0	0	ND	ND	–	–
**Pb**	ND	ND	ND	ND	ND	21/4	3/8	–	–
**Bi**	0	0	0	0	0	0/0	0/0	–	–
**U**	ND	0	ND	ND	ND	ND	ND	–	< 0.2

Elemental mass fractions determined by semi-quantitative analysis by ICP-MS (μg/g particle) (ND = not detectable). Blank concentrations were subtracted. NIST2975 and CB were analyzed in duplicates (separated by slash).^a^Results previously published in Bendtsen et al. (2019) [12]. ^b^Reference values from Ball et al. (2000) [33] (the study only analyzed Co, Cu, Fe, Ni, V, and Zn). Note that we extracted for significantly longer time (several days vs. overnight) and with 25% nitric acid instead of 0.1 M phosphate buffer. ^c^Reference values from the MAK-Collection for Occupational Health and Safety (written communication of unpublished data of Degussa) [34]

#### Content of polycyclic aromatic hydrocarbons (PAHs) in the collected particles

The samples were analyzed for native PAHs and PAH derivatives by gas chromatography–mass spectrometry (GC-MS). In total, particle extracts were analyzed for 20 native PAHs, 13 alkylated PAHs (alkyl-PAHs), 14 nitrated PAHs (nitro-PAHs), 10 oxygenated (oxy-PAHs) and 6 dibenzothiophenes (DBTs). Table 3 shows the total amount of different groups of PAH derivatives in μg per g collected particle mass (PM). Values for the individual compounds are given in Additional file A. DEP9.7 showed the highest mass fractions of native PAHs, which was expected due to the low temperature combustion mode caused by the lower intake O_2_ concentration. DEP9.7 also contained the highest levels of nitro-PAHs.

**Table 3:** Summary of PAH content (μg/g) in the PM samples. A full list of all PAH derivatives can be found in Additional file A

Particles	DEP			HVO	RME	*NIST2975*
Fuel	MK1 low sulfur diesel			Hydrotreated vegetable oil	Rapeseed methyl ester	
Intake O_2_%	**9.7**	**13**	**17**	**13**	**13**	**–**
Native PAHs	23,700	2470	858	9960	1180	52
Alkyl-PAHs	400	483	77	644	150	7
DBTs	47	78	128	86	94	10
Nitro-PAHs	131	21	8	65	40	34
Oxy-PAHs	2490	1450	314	2630	596	265
**Total PAHs**	26,800	4500	1390	13,400	2060	369
BaPeq^a^ (μg/g)	4685	165	59	1067	60	3

^a^Sum of BaPeq for 12 PAH (see Additional file L) out of in total 63 different measured PAHs and PAH derivatives

The highest level of the sum of all PAHs were found in DEP9.7, followed by HVO13, DEP13, RME13 and DEP17. The PAH content decreased with increased intake O_2_ for DEP9.7, DEP13, and DEP17, which agrees well with a more complete combustion at higher intake O_2_ concentration.

Compared to DEP13, HVO13 particles contained higher amounts of all PAHs, especially native PAHs and oxy-PAHs, while RME particles contained lower mass fractions of total PAHs.

The levels of DBTs followed a different trend than the other PAH derivatives, by increasing with increasing intake O_2_ concentrations. DBT levels were highest for DEP17, followed by DEP13 and DEP9.7, while DBT levels were similar for DEP13, HVO13 and RME13.

Levels of native PAHs and PAH derivatives analyzed by GC-MS analysis (μg/g particles). Particle samples, blank control filters and standard reference material NIST2975 were analyzed for 20 native PAHs, 13 alkylated PAHs (alkyl-PAHs), 14 nitrated PAHs (nitro-PAHs), 10 oxygenated PAHs (oxy-PAHs) and 6 dibenzothiophenes (DBTs). PAH concentrations of blank control filters were substracted. A full list of all PAH derivatives can be found in Additional file A.

#### Reactive oxygen species (ROS) generation

Reactive oxygen species generation by the five different particle samples and by CB was measured acellularly, where generated ROS causes formation of 2′,7′ dichlorofluorescein (DCF) from DCFH_2_ which can be spectrofluorimetrically measured. The initial slope of the curve (alfa values) of measured fluorescence of the five particles are given in Table 4. In comparison, the alfa value of CB was 41,554. ROS data were reported previously [31]. The ROS formation potential increased with the intake O_2_ concentration independently of fuel type. This indicates that engine operating conditions, combustion temperatures and the availability of O_2_ are important engine parameters that can alter the ROS formation potential of the soot [31].

**Table 4:** ROS generation (fluorescence per μg)

Particles	DEP			HVO	RME
Fuel	MK1 low sulfur diesel			Hydrotreated vegetable oil	Rapeseed methyl ester
Intake O_2_%	**9.7**	**13**	**17**	**13**	**13**
ROS (alfa)	2685	14,682	24,039	19,998	14,457

The alfa values represent the initial slope of the dose-response curve of measured fluorescence in relation to particle mass

#### Particle size distribution in dispersion

For the in vivo study, the diesel exhaust particles were collected on Teflon filters with a PM1 pre-separator, extracted using methanol, and dispersed in vehicle and diluted. The particles were dispersed in 0.1% Tween in Nanopure water and sonicated to achieve stable dispersions [35, 36]. The hydrodynamic number and intensity size distributions were measured by Dynamic Light Scattering (DLS). Similar distributions of particles sizes corresponding to agglomerates/aggregates were observed for the five particles, in the same size range as seen for CB and NIST2975 (Additional file B).

### Pulmonary exposure of C57BL/6 mice

Mice were exposed by intratracheal instillation to 6, 18, and 54 μg of dispersed particles and euthanized on day 1, 28 and 90. Exposure to the vehicle (Nanopure water with 0.1% Tween) was included as exposure control (*vehicle*). In addition, exposure to blank filter extraction dispersed in 0.1%Tween was included as blank filter extraction control (*extract*). Carbon black Printex90 particles dispersed in the same vehicle (0.1% Tween) were included at a single dose level as reference particle (CB) to enable comparison with previous studies [12, 35, 37–46].

#### Pulmonary histopathology

**Fig. 2:**
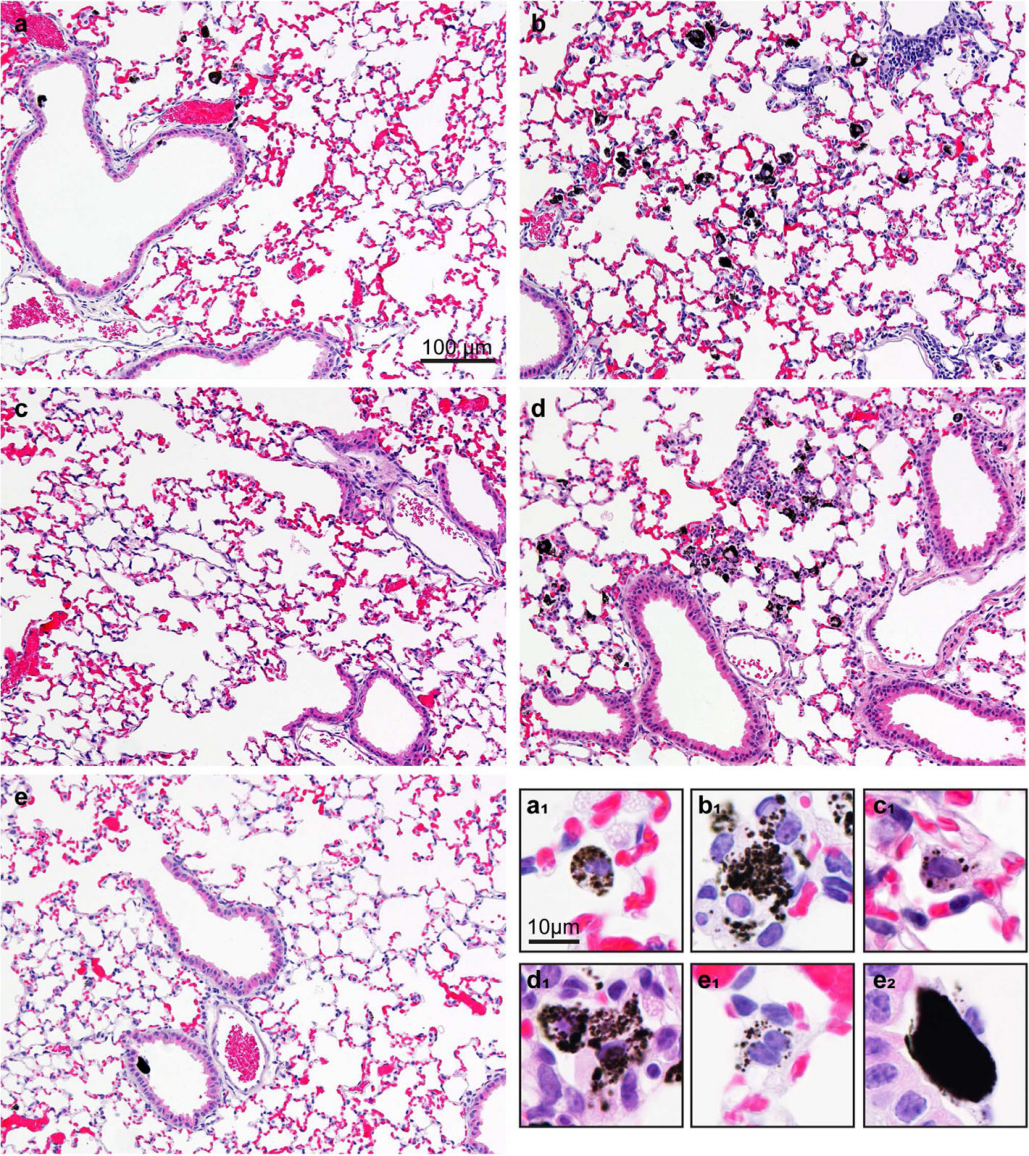
Mouse lung histology 28 days post-exposure to 54 μg DEP9.7 (**a**), DEP13 (**b**), DEP17 (**c**), and HVO13 (**d**) and RME13 (**e**). (a_1_)-(e_2_) are high magnification images of black particles in exposed lungs. Haematoxylin and eosin stained

Pulmonary histopathology was evaluated on day 28 (Fig. 2) and day 90 (not shown). Generally, only minor histopathological changes were observed. Most particle retention on day 28 was observed in DEP13 and HVO13 exposed mice (Fig. 2b, d). Histopathological changes observed for these particles were related to macrophage and lymphocyte infiltration. For DEP9.7, DEP17 and RME13, particles were scarce and no apparent histological changes were observed (Fig. 2a, c, e). All five particle types appeared as black micron-sized agglomerates mainly phagocytized in macrophages (Fig. 2 a_1_-e_1_). In addition, some larger dense aggregates were observed for RME13 (Fig. 2 e_2_).

#### Cell composition in bronchoalveolar lavage (BAL) fluid

**Fig. 3:**
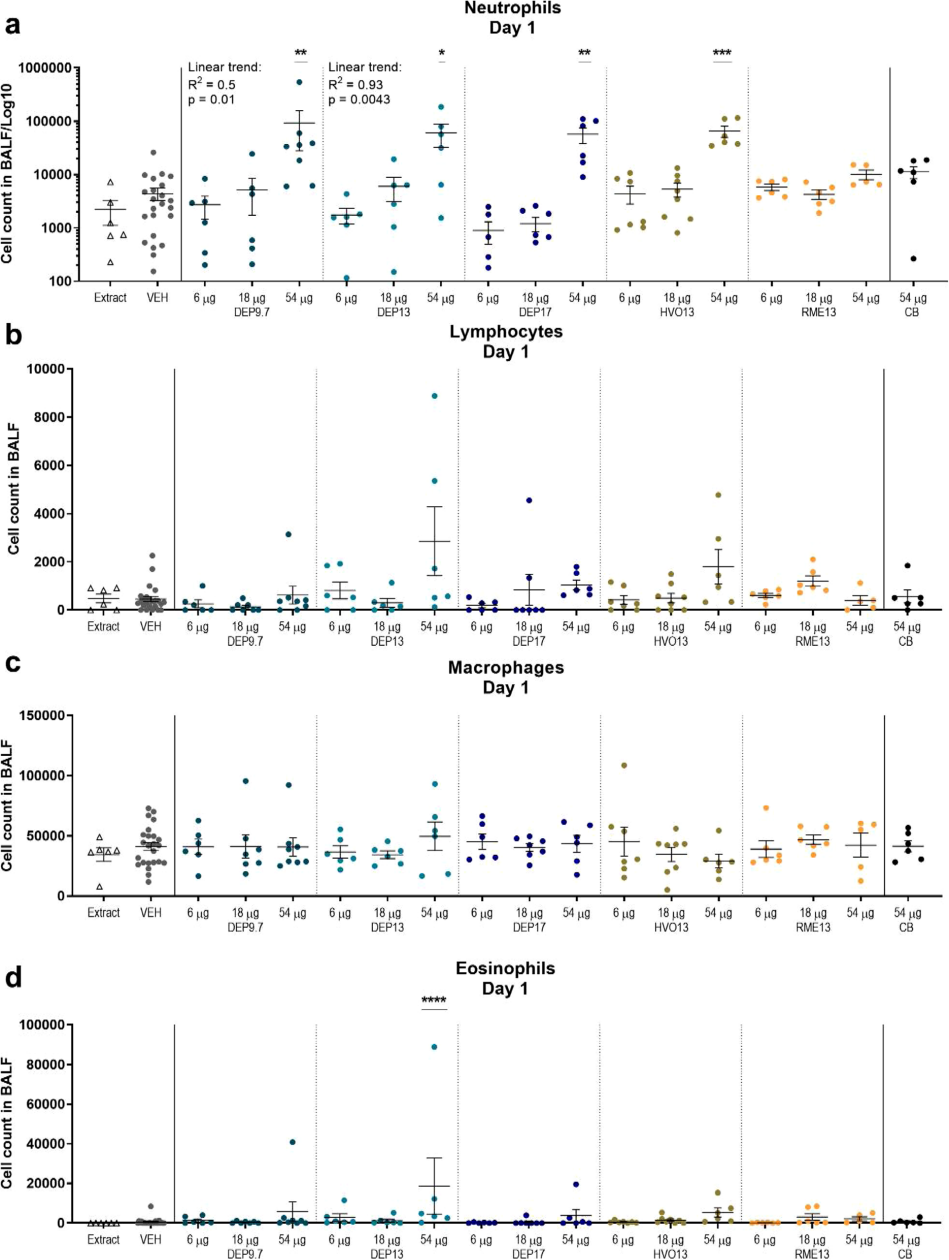
Cell counts of broncho-alveolar lavage of mice day 1 post-exposure to 6, 18, and 54 μg DEP9.7, DEP13, DEP17, HVO13 and RME13; **a**) Neutrophils. Four values of zero were excluded due to the log10 axis chosen to depict the large response range. **b**) Lymphocytes, **c**) Macrophages, **d**) Eosinophils. * = *p* < 0.05. ** = *p* < 0.01, *** = *p* < 0.001, **** = *p* < 0.0001

**Fig. 4:**
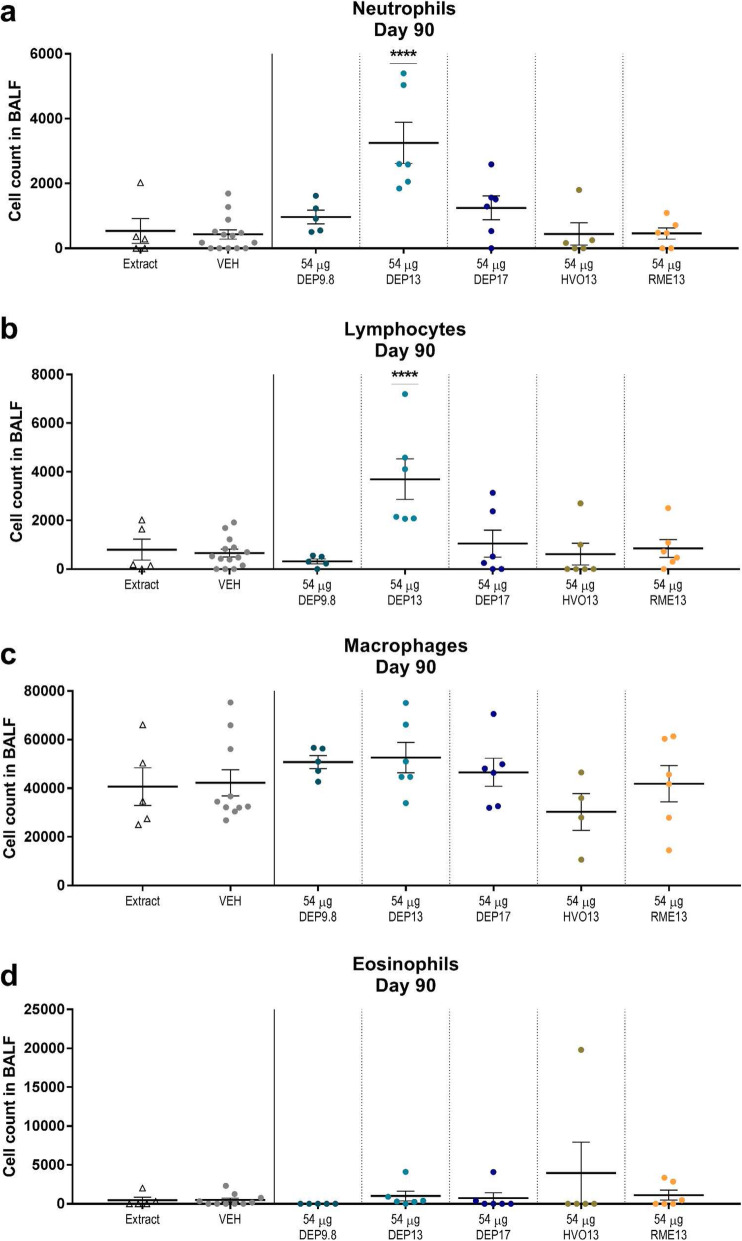
Cell counts of broncho-alveolar lavage of mice day 90 post-exposure to 54 μg DEP9.7, DEP13, DEP17, HVO13 and RME13; **a**) Neutrophils, **b**) Lymphocytes, **c**) Macrophages, **d**) Eosinophils. * = p < 0.05. ** = p < 0.01, *** = p < 0.001, **** = p < 0.0001

Pulmonary inflammation was evaluated 1, 28 and 90 days post-exposure by differential cell count of BAL fluid cell composition (Figs. 3 and 4, and Additional files C and D).

##### Day 1 post-exposure

Neutrophil influx was significantly increased in mice exposed to 54 μg of DEP9.7, DEP13, DEP 17, and HVO13 compared to vehicle control with similar, significant dose-response relationships. The 54 μg doses exceeded the level of response to the CB reference at the same dose level. DEP9.7 exposure induced high response (Fig. 3 a). In contrast, RME13 did not cause significantly increased neutrophil influx as compared to vehicle. No consistent differences were found for lymphocytes and macrophages compared to vehicle (Fig. 3 b and c). For eosinophils, only DEP13 at 54 μg significantly increased the influx compared to vehicle (Fig. 3 d).

##### Day 28 post-exposure

On day 28, some particle exposures seemingly resulted in reverse dose response relationships for neutrophil influx, with significant increase for 6 μg of RME13 and DEP13 compared to vehicle (Additional file, D 1). For 18 μg DEP13, there was a very low response, even significantly decreased compared to vehicle mice. CB exposure was not statistically different from vehicle for neutrophils. However, lymphocytes were significantly increased for CB exposed mice compared to vehicle (Additional file, D 2). No statistical differences were found for macrophages and eosinophils (Additional file, D 3 and 4).

##### Day 90 post-exposure

On day 90 following exposure, DEP13 had a significantly increased numbers of neutrophils and lymphocytes compared to vehicle (Fig. 4 a and b). Presence of macrophages was also observed for all exposed mice at levels around 40,000 cells, including vehicle mice, where HVO13 had a noteworthy lower cell numbers (Fig. 4 c). No statistically significant differences were seen for eosinophils (Fig. 4 d).

#### Serum amyloid A in lung

##### Day 1 post-exposure

**Fig. 5:**
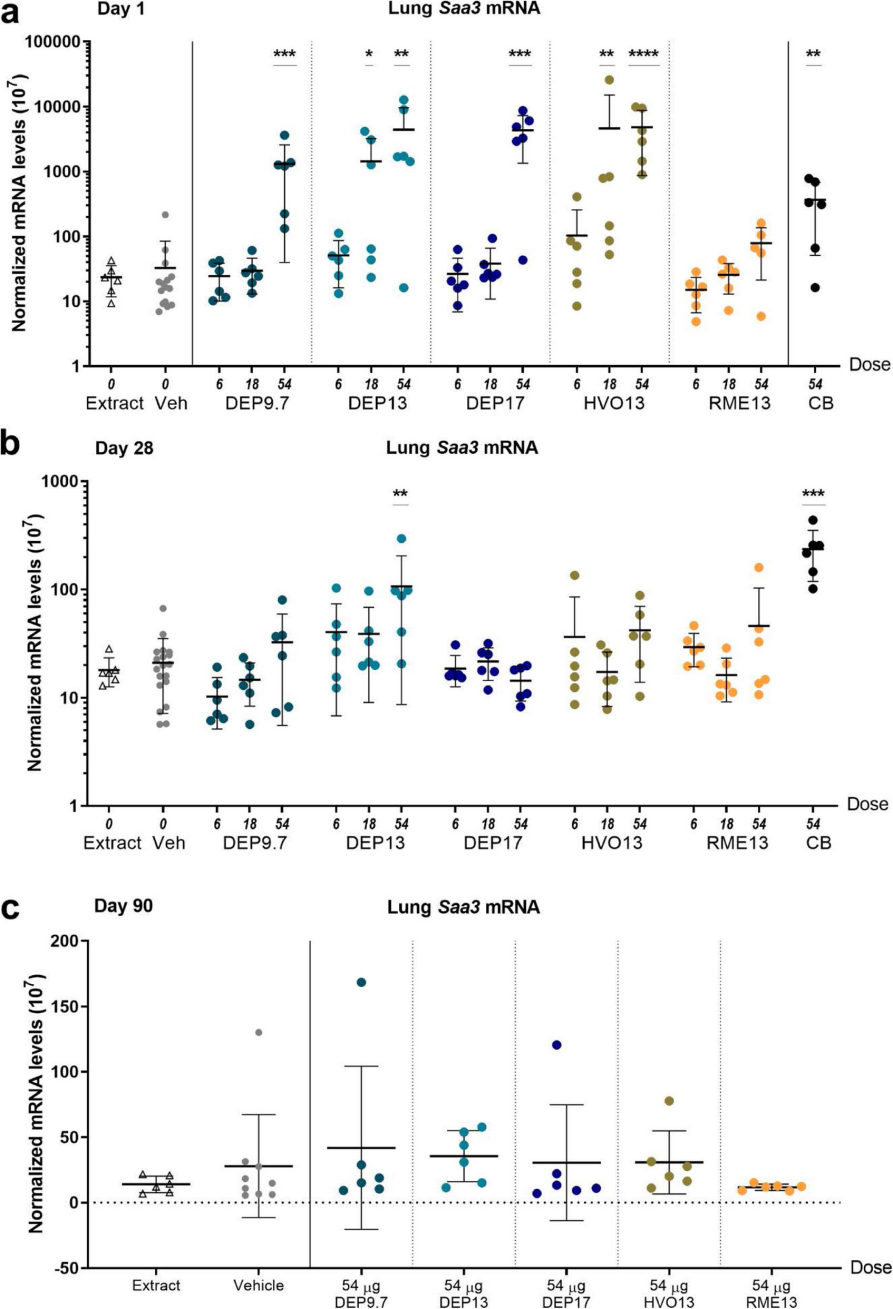
*Saa3* mRNA levels in lung tissue of mice day 1 (**a**), 28 (**b**) and 90 (**c**) post-exposure to 6, 18 and 54 μg DEP9.7, DEP13, DEP17, HVO13 and RME13. * = p < 0.05. ** = p < 0.01, *** = p < 0.001, **** = p < 0.0001

*Saa3* mRNA levels were used as biomarker of acute phase response [20, 46, 47] in lung tissue. On day 1, significant, dose-dependent increase in *Saa3* mRNA levels compared to vehicle was observed in lung tissue for all exposures, except for RME13 (Fig. 5 a). DEP9.7 (*p* < 0.0003), DEP13 (p < 0.0003), DEP17 (p < 0.0003), and HVO13 (*p* < 0.0001) of 54 μg all exceeded the level of CB. DEP13 (*p* = 0.0110) and HVO13 (*p* = 0.0074) of 18 μg were also significantly increased compared to vehicle. *Saa3* mRNA levels in lung correlated well with neutrophil influx (R^2^ = 0.5902, *p* = 0.0002) (Additional file E).

Test for linear dose-response was significant for all exposures, except for RME13 (Fig. 5 a).

##### Day 28 and 90 post-exposure

On day 28, DEP13 of 54 μg (*p* = 0.0034) and CB (*p* = 0.0007) were still increased compared to vehicle (Fig. 5 b). No significant differences were seen on day 90 (Fig. 5 c).

#### DNA damage

**Fig. 6:**
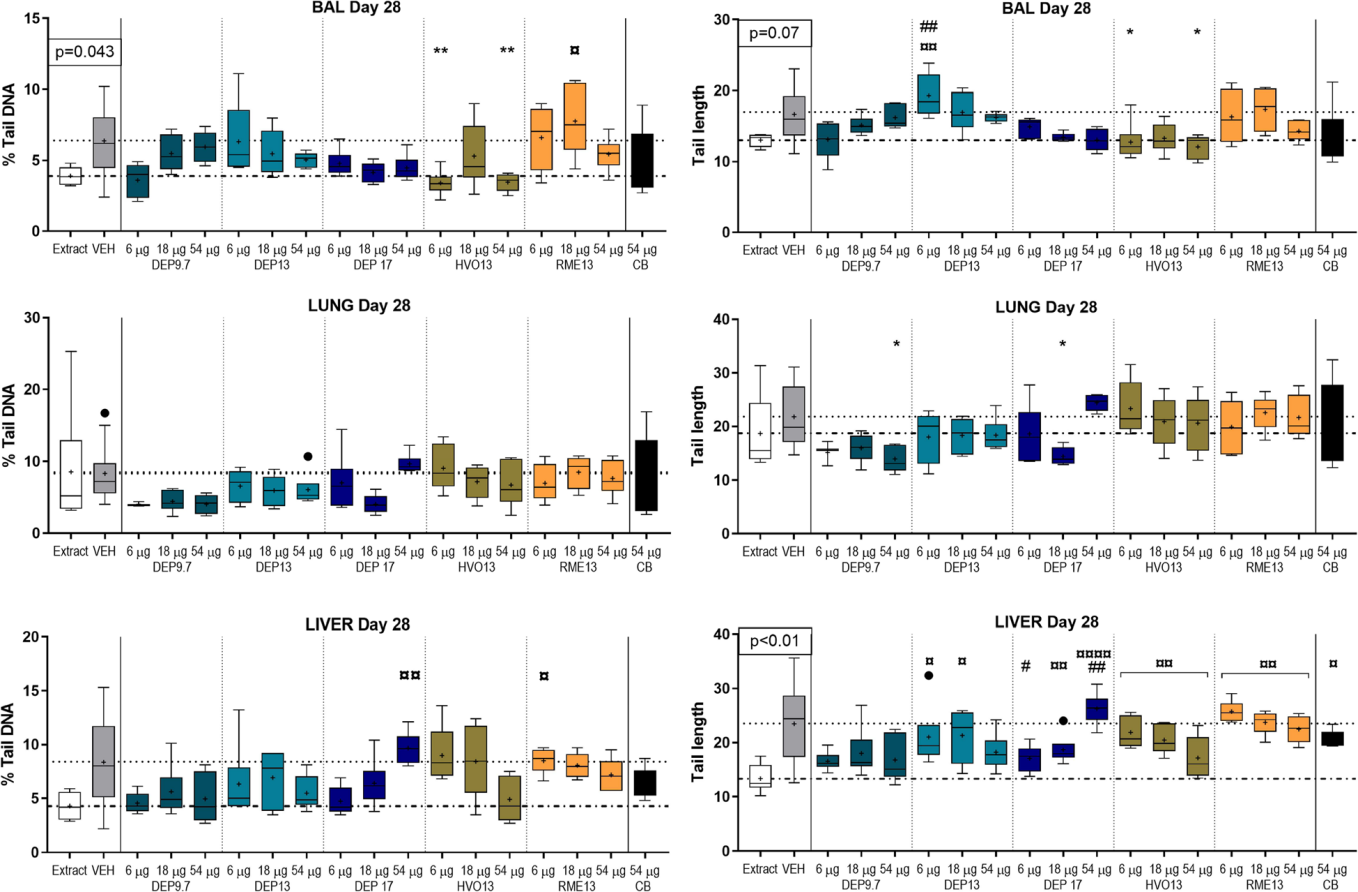
DNA strand breaks assessed in the Comet assay by %Tail DNA and Tail length in bronco-alveolar lavage cells, and lung and liver tissue of mice day 28 post-exposure to 6, 18 and 54 μg DEP9.7, DEP13, DEP17, HVO13 and RME13. ¤ = compared to extract, * = compared to VEH, ## = compared to CB. Dot-dash line: extraction control level, dotted line: vehicle control level

**Fig. 7:**
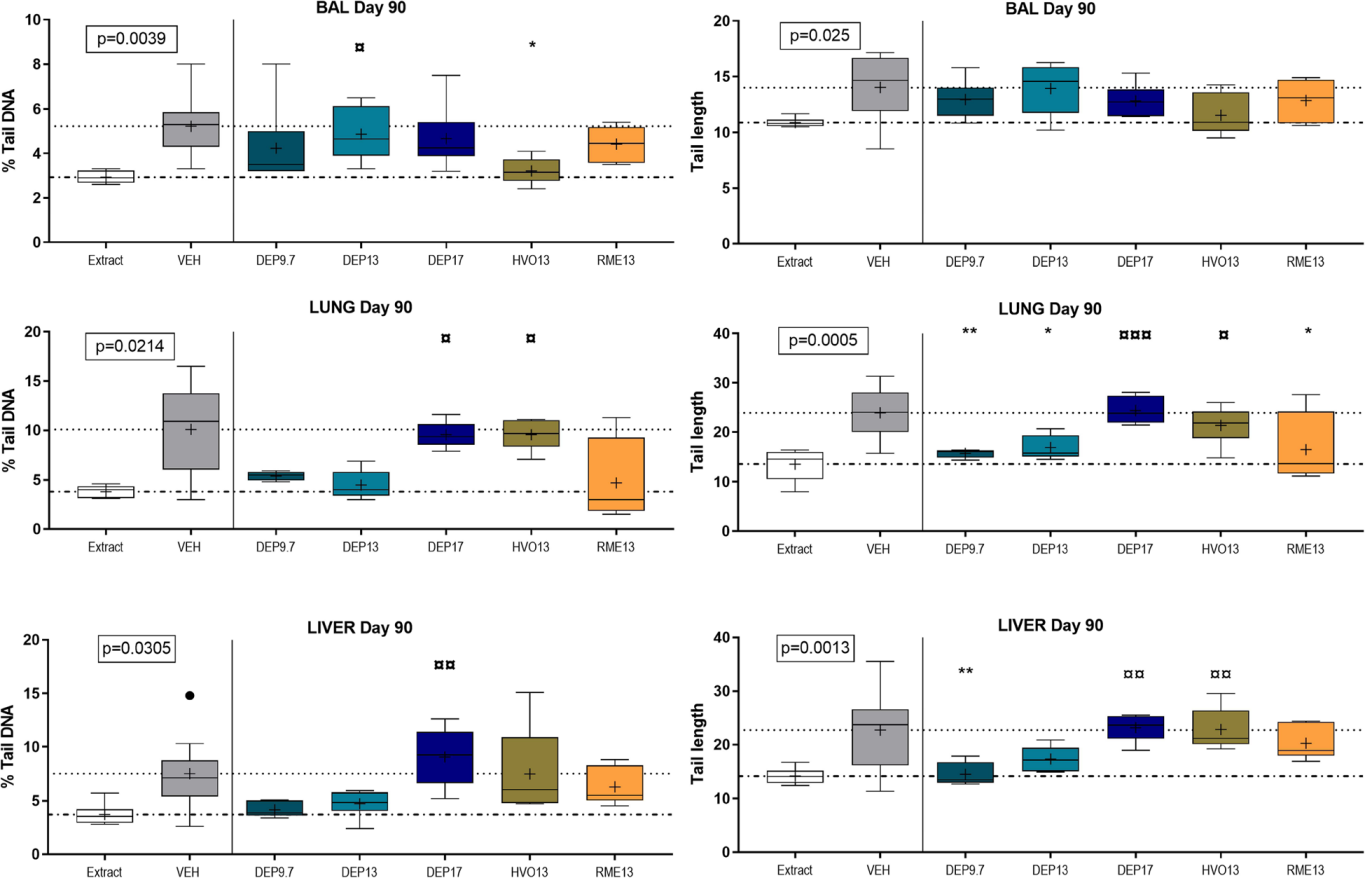
DNA strand breaks assessed in the Comet assay by % Tail DNA and Tail length in bronco-alveolar lavage cells, and lung and liver tissue of mice day 90 post-exposure to 6, 18 and 54 μg DEP9.7, DEP13, DEP17, HVO13 and RME13. ¤ = compared to extract, * = compared to VEH, ## = compared to CB. Dot-dash line: extraction control level, dotted line: vehicle control level

Genotoxicity was evaluated as DNA strand break levels in the comet assay, using comet tail length and % tail DNA in BAL derived cells, lung cells and liver cells on day 1, day 28 and day 90 post-exposure (Figs. 6 and 7, and Additional file F). Generally, variations were observed between exposures and within the vehicle control groups across doses and time points. No increases in DNA stand break levels were observed as compared to vehicle. There were no differences on day 1 (data not shown). There was a significant difference between vehicle and blank filter extraction control, especially for liver on day 28 (*p* = 0.0023), and for all tissues on day 90 (*p*-values: 0.0005–0.0305), with blank filter extraction control samples having significantly lower DNA strand break levels compared to vehicle (Figs. 6 and 7).

When compared to the blank filter extraction control, DEP13 at 6 μg was increased on day 28 for tail length in BAL cells (*p* = 0.078). For tail length in liver cells on day 28, RME13 (*p* = 0.0017), HVO (*p* = 0.004), DEP13 (6 and 18 μg: *p* = 0.0480), DEP17 (18 μg: *p* = 0.0072; 54 μg: *p* < 0.0001), and CB were increased (*p* = 0.0405) (Fig. 6). On day 90, DEP17 and HVO13 were increased in both lung (DEP17: *p* = 0.0008; HVO13: *p* = 0.0187) and liver cells (DEP17: *p* = 0.0026; HVO13: *p* = 0.0062) compared to blank filter extraction control (Fig. 7).

### Correlations

Linear regression analyses were carried out in order to assess physicochemical properties as predictors of inflammation and acute phase response. For this, the SSA of CB was estimated to 230 m^2^/g, although other values have been reported [5, 11, 48].

**Fig. 8:**
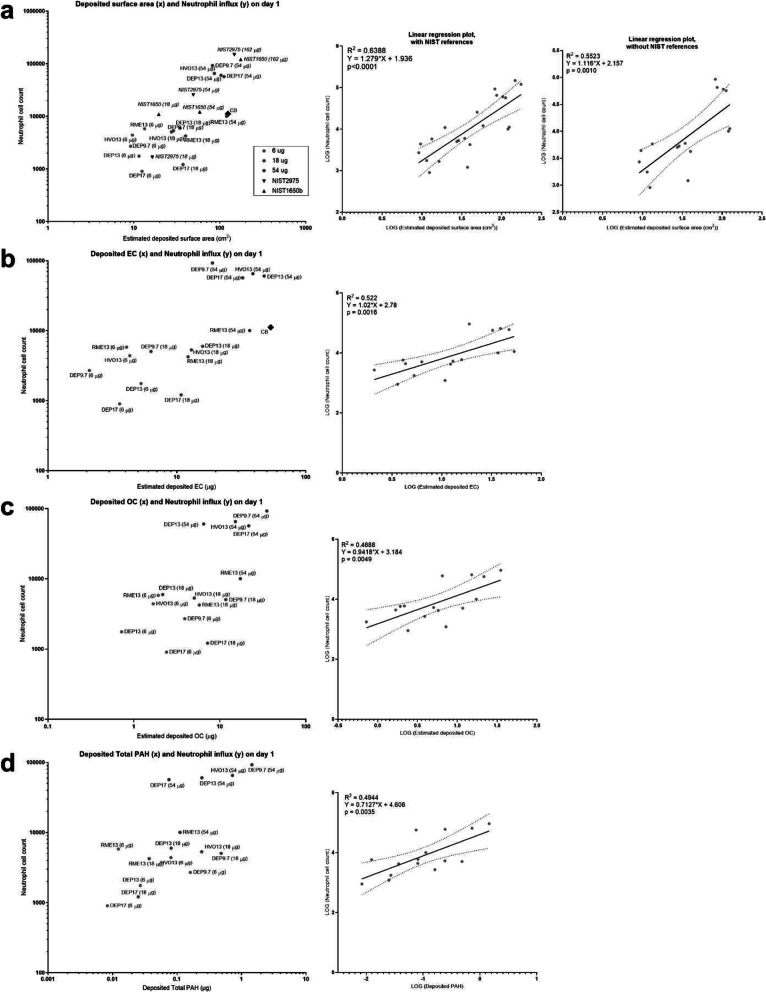
Deposited surface area, EC, OC, and PAH correlations with neutrophil influx on day 1. (**a**) Estimated deposited surface area, original data (left panel) and linear regression plots with and without NIST references (right panels). (**b**) Estimated deposited elemental carbon, original data (left panel) and linear regression plot (right panel). (**c**) Estimated deposited organic carbon, original data (left panel) and linear regression plot (right panel). (**d**) Estimated deposited Total (native) PAH, original data (left panel) and linear regression plot (right panel). The estimated deposited specific surface area (SSA), elemental carbon (EC), organic carbon (OC) and PAHs were calculated by multiplying the physicochemical values by the dose (EC and OC: dose in μg * EC fraction = deposited EC in μg; for SSA: dose in g * SSA m^2^/g = deposited SSA in g; for PAH: dose in g * PAH μg/g = deposited PAH in μg). The data was log-transformed for the linear regression analysis

Neutrophil influx correlated well with estimated deposited SSA on day 1 (Fig. 8 a), where 50–60% of the variation in neutrophil influx could be explained by estimated deposited SSA. To compare with known reference particles, the plot was made with either inclusion (R^2^ = 0.6388, p < 0.0001) or exclusion (R^2^ = 0.5523, *p* = 0.0010) of previously published data on surface area and neutrophil influx on standard reference material (SRM) NIST2975 and NIST1650, which are diesel exhaust particles derived from a diesel-powered industrial forklift and a heavy duty truck, respectively. The vehicle used for these historical data was Nanopure water, without the addition of Tween80 [12]. In a previous study comparing the effect of vehicle on carbon black-induced neutrophil influx, there was no differences between 0.1%Tween80 and Nanopure water [35]. Similar significant correlations with neutrophil influx, with 40–50% of the variation explained, were seen when deposited elemental carbon (EC), organic carbon (OC) and total PAH dose were used as dose metrics (EC: R^2^ = 0.522, *p* = 0.0016; OC: R^2^ = 0.4688, *p* = 0.0049), Total PAHs: R^2^ = 0.4944, *p* = 0.0035) (Figs. 8 b-d). Deposited metals (sum of most abundant metals measured) were poorly correlated with neutrophil influx on day 1 (Additional file G a).

**Fig. 9:**
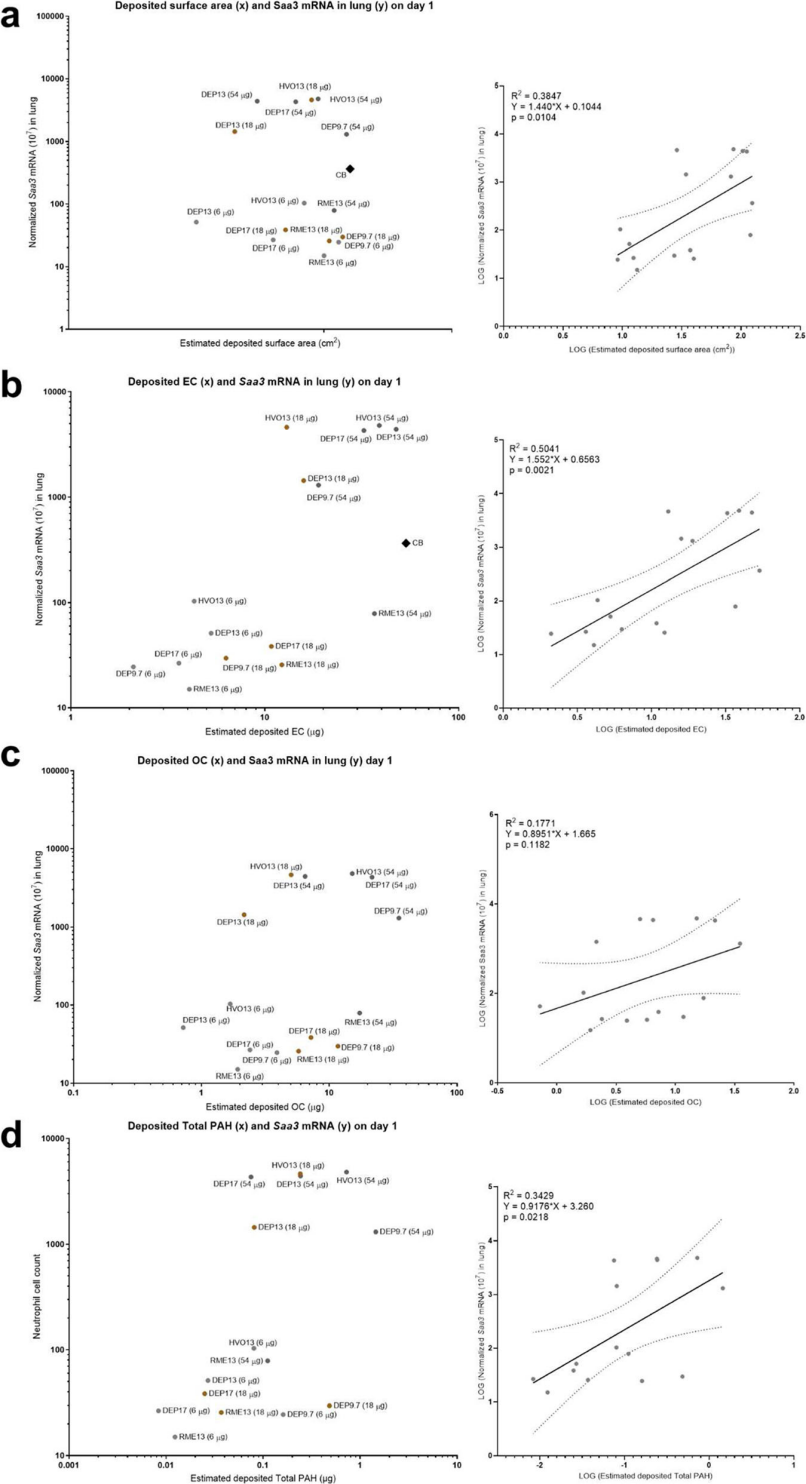
Deposited surface area, EC, OC, and PAH correlations with *Saa3* mRNA on day 1. (**a**) Estimated deposited surface area, original data (left panel) and linear regression plot (right panel). (**b**) Estimated deposited elemental carbon, original data (left panel) and linear regression plot (right panel). (**c**) Estimated deposited organic carbon, original data (left panel) and linear regression plot (right panel). (**d**) Estimated deposited Total (native) PAH, original data (left panel) and linear regression plot (right panel).The estimated deposited specific surface area (SSA), elemental carbon (EC), organic carbon (OC) and PAHs were calculated by multiplying the physicochemical values by the dose (EC and OC: dose in μg * EC fraction = deposited EC in μg; for SSA: dose in g * SSA m^2^/g = deposited SSA in g; for PAH: dose in g * PAH μg/g = deposited PAH in μg). The data was log-transformed for the linear regression analysis

*Saa3* mRNA levels in lung tissue on day 1 correlated well with deposited EC (R^2^ = 0.5041, *p* = 0.0021), but less with deposited surface area, OC, and PAHs (surface area: R^2^ = 0.3847, *p* = 0.0104; OC: R^2^ = 0.1771, *p* = 0.1182, PAHs: R^2^ = 0.3429, *p* = 0.0218) (Fig. 9 a-d).

As no significant differences were seen on day 28 for neutrophil influx and for genotoxicity on day 1, these data were not subjected to correlational analysis.

**Fig. 10:**
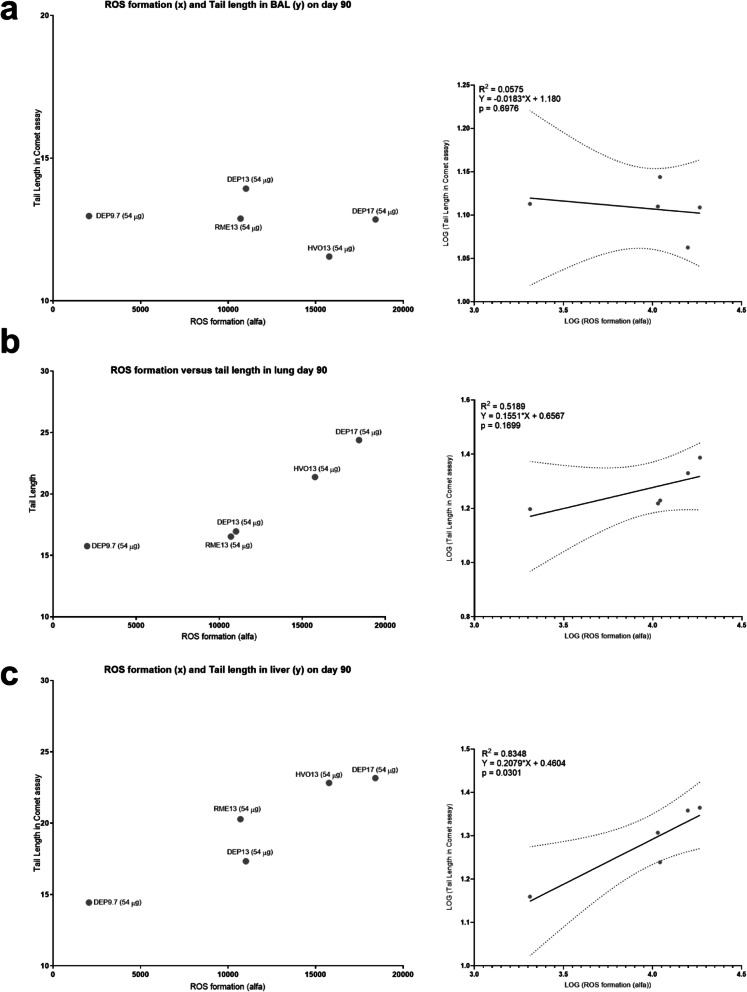
ROS formation correlations with Tail Length in Comet assay on day 90. (**a**) BAL, original data (left panel), linear regression plot (right panel). (**b**) Lung, original data (left panel), linear regression plot (right panel). (**c**) Liver, original data (left panel), linear regression plot (right panel). ROS is given in arbitrary alfa-values. Data were log-transformed for the linear regression analysis

On day 28, ROS formation correlated with genotoxicity in lung (R^2^ = 0.3476, *p* = 0.0162) (Additional file H), whereas Total PAHs (Additional file I) and BaPeq (not shown) did not correlate well with genotoxicity, measured as Tail length in BAL, lung and liver in the Comet assay. On day 90, Tail length did not correlate with Total PAHs (Additional file J), or BaPeq (not shown) (Fig. 10 a-b). However, ROS correlated well with Tail length in the liver (Fig. 10 c) (R^2^ = 0.8348, *p* = 0.0301). On day 90, neutrophil influx did not correlate with surface area or deposited elemental carbon (Additional file G b-c).

## Discussion

Five diesel exhaust particles were designed to differ in regards to chemical and physical properties in order to assess the toxicity of the different particle properties. Toxicity was evaluated in terms of inflammation, acute phase response, DNA strand breaks and histopathological changes.

### Physicochemical properties

#### Organic and elemental carbon, primary particle size and surface area

A qualitative overview of the physicochemical properties and the toxicity parameters for the five different particle samples are shown in Table 5.

**Table 5:** Qualitative relative comparison of data on particles from combustion of three fuel types

Physical-chemical properties	DEP			HVO	RME
	MK1 low sulfur diesel			Hydrotreated vegetable oil	Rapeseed methyl ester
Intake O_2_%	**9.7**	**13**	**17**	**13**	**13**
Oxygen content in fuel	0	0	0	0	10%
PAHs	+++	+	−/+	++	−/+
OC	+++	+	++	++	++
EC	+	+++	+	++	++
ROS	+	++	+++	+++	++
Metals	++	+	+++	++	++
Surface Area	++	++	++	++	+++
**In vivo response**					
Histopathology (lung)	−/+	++	−/+	++	−/+
Material presence	+	++	+	++	+
Large agglomerates	–	–	–	–	+
Inflammation as neutrophils and lymphocytes in BAL fluid	+	++	+	+	−/+
Eosinophil response	–	+	–	+	–
Inflammation as acute phase response	++	+++	++	+++	–
DNA damage	–	+	++	++	+

*PAHs* Polycyclic aromatic hydrocarbons, *OC* Organic compounds, *BC* Black carbon, *ROS* Reactive oxygen species, *BAL* Bronco-alveolar lavage. Relative categories: “-”: not present, “-/+” = very low content/response, “+” = low content/response, “++” = medium content/response, “+++” = high content/response.

The obtained variations in the physical properties (primary particle size: 15–22 nm, SSA 152–222 m^2^/g and count median mobility diameter: 55–103 nm) were moderate, but are within the variations observed for the accumulation mode of real-world engine out emissions [49–51]. The renewable fuels reduced the PM1 emissions and the particle mobility size, which also have been found in previous studies [52–54].

Large variations were obtained for the organic carbon fraction (0.12–0.60), PAH fraction (1–27 μg/mg) and metal contents (0.9–16 μg/mg).

The combustion conditions strongly affected the exhaust emission composition. The particles generated at 13% intake O_2_ concentration had the highest EC fraction, while higher and lower intake O_2_ concentration increased the OC fraction. Organic carbon emissions in diesel exhaust are mainly lubrication oil-derived under lower/medium EGR (higher intake O_2_ concentrations) [51] and can therefore be expected to show less dependence on fuel type. This can also be seen as the renewable fuels had ~ 2.8 times lower PM1 mass emissions and a factor ~ 2.3–2.8 higher OC/EC fraction (Table 1). These observations are consistent with previously reported reduced EC/BC emissions from renewable diesel fuels [53].

The sample generated at low intake O_2_ concentration (DEP9.7) had the highest PAH mass fraction as a consequence of a higher degree of incomplete combustion process as the oxygen availability and combustion temperature were decreased [55].

All samples, except DEP9.7, were potent ROS producers, however all exhibited lower ROS formation potential per SSA than CB. The organic compounds were hypothesized to reduce the ROS formation compared to pure EC surfaces by reducing the physical particle size by filling pores in the soot agglomerate and/or by shielding of active solid soot surfaces [31]. Gren et al. [31] dentified that the ROS potential per mass was highest for DEP17 and lowest for DEP 9.7 and correlated with increasing combustion temperature. In addition, a higher ROS formation potential was associated with an increasing refractory O:C ratio from aerosol mass spectrometry. These two observations suggested that increasing combustion temperature and O_2_ availability are linked to (1) more graphitic soot structures, and (2) increasing partial oxidation of soot surfaces. In turn, these processes appear to be strongly linked to the ROS formation potential [31] and, in this study, to particle-induced DNA strand-breaks.

#### Metals

The most abundant metals were Cu and Fe, with these and several other trace elements were increased 5–17 fold in DEP17 compared to the other four samples. Similarly, the metal fraction was 2.5–3.5 times higher for both renewable diesel fuels compared to DEP13. Increased concentrations of Cu and Fe in the used lubricating oil was found in a previous study using the same engine setup [56]. The emissions of metals (μg/m^3^ in undiluted exhaust) are within a factor 2 for all samples, but as the PM emission was much lower for DEP17, and lower for HVO13 and RME 13, compared to DEP13, the relative metal fraction of the total collected PM was increased. The Zn and Mg measurements were excluded from the data set, because very high concentrations were detected in the blank filter control samples. As we only included one blank sample in the analysis, it was not possible to conclude whether the Zn and Mg came from the particles or were the result of background contamination. In turn, **s**everal of the constituents of lubrication oil and diesel particles were not measured, including P and Ca, although the mass percentages of these would be expected to be low.

#### PAHs

The combustion of all three fuels resulted in significant particulate PAH emissions. The PAH emissions in this study were primarily combustion-derived, since HVO and RME have essentially no aromatic components in the fuel. The PAH fraction of the total particle mass was substantially higher for HVO13 than for DEP13. This may partly be explained by higher PM1 emission levels from DEP13 compared to HVO13. RME13, the emissions from combustion with the oxygenated biofuel RME, contained lower amounts of PAHs compared to both DEP13 and HVO13. Oxygen containing biodiesel, such as RME, has previously been found to reduce PAH emissions [57, 58]. The cause of this relation is not clear, but the oxygen available in the fuel may influence both the soot formation and its precursors (of which PAHs are major components) as well as the oxidation (i.e., in combustion removal) of soot and PAHs. As combustion temperatures were reduced with the addition of EGR (decreased intake O_2_), the PAHs/PM1 and PAHs/EC ratios increased strongly. It is interesting to note that the ratio of BaPeq compared to the total PAHs are higher for DEP9.7 compared to the other samples. This can indicate that the low temperature combustion favors the formation of larger and more genotoxic PAHs. Nonetheless, the main parameter driving the sum of benzo(a)pyrene equivalents (BaPeq) was the total sum of emitted PAHs (see Table 3 and Additional file L), and not differences in the PAH composition.

### Toxicity

Inhalation is the golden standard for testing pulmonary toxicity, whereas pulmonary intratracheal instillation allows control of the deposited dose, and is thus suitable for comparison of hazard potential between different particles. We used 0.1% Tween as vehicle [35, 59], since all five diesel exhaust particle samples could be dispersed using this vehicle. Furthermore, two different control groups were included, vehicle controls and blank filter extraction controls.

#### Histopathology and particle retention

Black particles compatible with DEP were readily observed in lungs 28 days post-exposure to DEP13 and HVO13. In RME13-exposed lungs, some large aggregates of particles were observed, but overall there seemed to be less particles present at day 28 in RME13, DEP9.7 and DEP17 exposed lungs than in DEP13 and HVO13. As the amount of particles appeared to be greatest for the two DEP types with the most pronounced inflammatory response, differences in biopersistence of the particles could potentially explain the observed differences in pulmonary responses at day 28. Unfortunately, we do not have data to address this further in the current study, but DEP13 and HVO13 were the two diesel exhaust particles with highest content of EC, reflecting the insoluble carbon core. However, RME13 was similar in OC/EC ratio to DEP13 and HVO13, and if the response was dependent on the EC content, we would expect a similar response. There could be a difference in the EC nanostructure for RME particles, considering the content of 10% O_2_ in the fuel itself. This might change the combustion characteristics and the soot oxidation, and previous studies have reported different nanostructures depending of renewable diesel fuels compared to diesel [60, 61].

#### Inflammation and acute phase response

The total deposited surface area is an important predictor of neutrophil influx into the lung for insoluble particles [19]. We found dose-dependent inflammation for most of the studied particles at day 1. Interestingly, RME13 did not induce inflammation in terms of neutrophil or lymphocyte influx at the assessed dose levels despite having similar SSA as the other particles. In addition, DEP13 appeared to be more inflammogenic than the other diesel exhaust particles, as increased influx of neutrophils and lymphocytes was still observed 90 days post-exposure. At day 28, no dose-response relationship was observed. This could be due to the low level of inflammation caused by the relatively low dose levels used in the current study. The standard diesel exhaust particles NIST2975 and NIST1650b did not induce neutrophil influx at 54 μg/mouse 28 days post-exposure in similar experimental set-ups [12, 62].

Acute phase response is the systemic response to various types of insults including bacterial infections, virus, infarction and all types of chronic inflammatory conditions [63], and acute phase response is a risk factor for cardiovascular disease in prospective epidemiological studies [64]. The acute phase protein serum amyloid A is causally related to atherosclerosis by promoting formation of atherosclerotic plaques [65]. We have previously shown that inhalation and pulmonary exposure to particles induce a dose dependent acute phase response [10, 20, 66], and we use *Saa3* mRNA levels as a biomarker of pulmonary acute phase response [20]. All the diesel exhaust particles except RME13 induced increased *Saa3* mRNA levels in the lung at day 1, and DEP13 even induced increased *Saa3* mRNA levels at day 28 alongside CB. S*aa3* mRNA levels have been shown to correlate closely with neutrophil influx and deposited surface area for insoluble particles such as CB and TiO_2_ nanoparticles and carbon nanotubes [10, 20, 44, 46]. We have previously shown that pulmonary exposure to NIST2975 induced pulmonary *Saa3* expression as well as increased plasma levels of SAA3 [12] and that pulmonary *Saa3* expression correlate with plasma SAA3 protein levels [67]. In the current study, *Saa3* mRNA levels correlated closely with neutrophil influx and deposited EC, but poorly with estimated deposited surface area on day 1 post-exposure. This could suggest that other particle components affecting the nanostructure in relation to the organic carbon content, in addition to SSA, are important contributors to the acute phase response of diesel exhaust particles. The acute phase response is an important cardiovascular disease risk factor [47], and inflammation may be linked to other pathologies including fibrosis [68] and secondary genotoxicity [69, 70].

#### DNA damage and ROS generation

The comet assay detects single strand breaks in DNA, and these can be caused by bulky PAH-DNA adducts and by particle-induced oxidative DNA damage. DNA damage in BAL and lung tissue may be caused by particles, metal ions, or PAHs released from the particles. DNA damage in the liver may be caused by primary genotoxicity caused by translocated particles [22] and by PAHs released into systemic circulation [71, 72]. In the current study, particle-induced DNA strand break levels in the liver on day 90 and in lung on day 28 correlated with particle-induced ROS, whereas PAH levels did not correlate with DNA strand break levels. The correlation between ROS and DNA damage observed for liver tissue was good (R^2^ = 0.8348), whereas the correlation for lung tissue depends heavily on a single data point for CB. However, both the ROS forming potential and the genotoxic effects of CB in vivo have been reported many times [5, 22, 37, 73]. Carbon black nanoparticles did not induce DNA strand breaks in the current study, but we have previously found CB-induced DNA strand breaks in BAL, lung and liver following inhalation and instillation [5, 22, 36, 44, 62, 74, 75]. We used 0.1%Tween80 as vehicle, since not all the DEPs could be dispersed satisfactory in pure water or 2% serum, which are the preferred vehicles [12, 40–45]. We have previously compared the effect of vehicle on CB-, TiO_2_- and CNT-induced toxicity [35]. CB-induced inflammation and genotoxicity in terms of DNA strand breaks in BAL cells were assessed for CB dispersed in pure water, 2% serum, 0.05% serum albumin, 10% BAL in saline, 10% BAL in water and 0.1%Tween80. The inflammatory response of CB was vehicle-dependent, but was numerically similar when CB was dispersed in pure water or in 0.1% tween. CB-induced DNA strand breaks in BAL cells were detected when CB was dispersed in pure water, 2% serum, 10% BAL in saline and 10% BAL in water, but not when CB was dispersed in 0.1%Tween80. It is therefore possible, that DEP-induced DNA strand breaks were underestimated in the current study.

When blank filter extraction control was used as reference, DEP13, DEP17, HVO13 and RME13 induced DNA strand breaks in lung and liver tissues. Particles with a diameter < 100 nm are able to translocate from the lung to the systemic circulation [76] and thus all DEP should be able to translocate from lung to systemic circulation. Subsequent accumulation in liver has previously been shown to occur for particles of similar size as the particles in the current study (approx. 20 nm), and particle-dependent ROS generation was suggested as the likely cause of the observed DNA strand breaks in liver following pulmonary exposure to carbon nanoparticles [22]. This suggests that carbon nanoparticles induce primary genotoxicity likely caused by ROS.

Carbon black nanoparticles are very efficient ROS generators [77] and induce oxidative DNA damage [37, 73] in vivo and in vitro. Carbon black nanoparticles and diesel exhaust particles have similar mutagenic potential in cell studies [16, 73] and diesel exhaust and carbon nanoparticles had similar carcinogenic potency in chronic inhalation studies in rats [78, 79]. This suggests that insoluble carbon nanoparticles such as the carbon core of diesel exhaust particles has the same mutagenic and carcinogenic potency as intact diesel exhaust. The present study supports the notion that particle-generated ROS contributes to the particle-induced genotoxicity.

#### Particle mass concentration and importance of fuel

The main effect of fuel type was that the PM emission rate in the undiluted exhaust was reduced by 65% for the two renewable diesels (HVO13 and RME13), compared to DEP13. RME13 clearly had less toxicological potential per mass for the endpoints included in this study, in terms of less inflammation and acute phase response, which are risk factors for cardiovascular disease. When taking into account the reduced PM1 emission factor, HVO also had a reduced toxicological response for most endpoints compared to MK1 diesel. Our results are in disagreement with the results by Brito et al. [57] and Shvedova et al. [58], who found that inhalation of soybean biodiesel exhaust increased inflammatory responses/cytokine secretion per mass compared to diesel in mice. Shvedova et al. [58] hypothesized that this is caused by increased oxidative stress caused by the organic matter, while we did not find any correlation with OC content.

Multiple animal studies (reviewed in [80]) have reported no difference in respiratory, cardiovascular and systemic response between biodiesel and petroleum diesel. Recently, Møller et al. [23] reviewed the available literature on cell and animal studies, and concluded that the evidence of biodiesel-induced inflammatory, oxidative stress and genotoxicity response is weak and inconsistent between studies. Furthermore, engine type, combustion conditions and after-treatment have been identified as major determinants of the chemical composition of the diesel exhaust particles [47, 81]. Thus, a major strength of the current study is that five different DEP were assessed in the same experimental setup, and that the three different fuel types were evaluated using the same engine, combustion conditions and after-treatment, thus ensuring comparability.

The mass emission should be considered when assessing the toxicity potential of the engine “output”, where reduced mass emission from the engine may lead to reduced toxicity potential of the engine output, even if the absolute toxicity per ng of particles is higher. In this study, the mice exposure dose levels varied by a factor of three. The PM emission factor for DEP13 was the same three-fold higher compared to RME13 and HVO13. Taking the PM emission factor into account, a comparison of engine emissions corresponds to comparing neutrophil influx following exposure to DEP13 at 54 μg and RME13 and HVO13 at 18 μg (Fig. 3 a), and this comparison shows that DEP13 induces more inflammation than RME13 and HVO13, which did not induce significantly increased neutrophil influx at 18 μg. However, a similar comparison for *Saa3* mRNA levels does not show as clear a picture.

In summary, exposure to particles from RME fuel with 13% intake O_2_ concentration resulted in the least inflammation and acute phase response, whereas the physicochemically similar HVO13 and DEP13 induced more inflammation and acute phase response. The small differences in the assessed physicochemical properties of RME13 compared to HVO13 and DEP13 cannot explain the lower inflammatory response. However, RME fuel contains oxygen that can alter the soot formation and oxidation processes, leading to different particle properties, which influence the particle toxicity. A change in particle toxicity may derive from either altered surface structure of the carbon core, for example in terms of the frequency of edge sites and the composition of strongly bound surface oxides, or by changes in the chemical composition of the organic components. A major part of the organic components is lubrication oil derived [31], but organic compounds derived from the combustion of the fuel are also emitted and may partition to the particle phase. Future studies can include direct measurements of the surface properties of collected samples, for example by X-Ray Photo Electron Spectroscopy. Future studies should also focus on understanding if the lower toxic effect of RME is valid under other driving conditions, such as with varied load and during real-world driving cycles. The extraction and re-dispersion procedure may also influence particle composition and future studies can investigate this further. DEP17 and HVO13 had the highest ROS production and 90 days post-exposure DNA strand breaks were increased in both lung and liver compared to blank filter extraction controls. The particle-induced ROS production correlated with DNA strand break levels in lung tissue on day 28 and liver tissue on day 90.

## Conclusions

We conclude that fuel type and combustion conditions are important factors for the physicochemical particle properties measured in this study, and that combustion conditions were more important than the fuel type. Among the tested fuels (Diesel, HVO, and RME), RME induced the least toxic response. We aimed at clarifying the relationship between physicochemical properties and toxicity. Estimated deposited surface area, elemental carbon, organic carbon, and PAHs correlated well with neutrophil influx, and ROS correlated with genotoxicity in lung tissue on day 28 and in liver on day 90. PAH content did not correlate with genotoxicity. Our study highlights specific surface area, elemental carbon content and ROS-generating potential as physicochemical predictors of diesel particle toxicity. Engine conditions that favor high combustion temperatures were found associated with higher ROS formation and more DNA strand breaks. These results may guide safe-by-design decisions for combustion engines.

## Material and methods

### Particle generation, collection and extraction

The particles were generated, collected and extracted as described in detail in Gren et al. [31], where the same particles are studied with other endpoints (in Gren et al. the particles are named slightly differently). Briefly, particles were generated with a modern heavy-duty diesel engine, operating on petroleum diesel (Swedish ultra low sulfur MK1) and two types of renewable diesels, hydrotreated vegetable oil (HVO; no aromatic and no oxygen content) and rapeseed methyl ester (RME, no aromatic but 10.6% oxygen) with no external exhaust after-treatment system. The engine was operated at a constant low load, and particle properties adjusted by varying the amount of exhaust gas recirculation (EGR). The amount of EGR changes the intake O_2_ concentration to the combustion cylinder, which in turn changes combustion temperature and thus the combustion conditions. An increase in EGR results in a decrease in intake O_2_ concentration and combustion temperature. In comparison to studies that generate particles from simulating realistic driving conditions, the advantage of our study design is the high control of combustion parameters that enable strong repeatability, and the possibility to link toxicological responses and particle physicochemical properties directly to combustion conditions.

The particles were collected on PTFE filters (Whatman PTFE, 150 mm, pore size 5 μm) using the final filter stage of a high volume cascade impactor (HVCI, BGI Inc.). The last impactor stage provided a cut-off size of 1 μm (PM1) for the filter sampling. The filters were soaked in 50 ml analytical grade methanol and sonicated (30 min, < 25 °C) three times. The extracts were pooled and dried with low-pressure evaporation (150 mbar, < 35 °C) in 10 mL glass vials [82]. A non-exposed PTFE filter was extracted with the same procedure and used as a “filter blank” reference sample. The gravimetric mass extraction efficiency was for all samples ≥85%. There was no clear trend in extraction efficiency for samples high in organic or elemental carbon (OC, EC) respectively. Furthermore, measured OC and EC mass fraction (%) were similar for air samples collected on quartz filters [31] and the PM extracted using methanol from the PTFE samples. This difference were within ±15% for all samples except DEP17 (± 25%). These minor differences might be due to uncertainties with gas phase OC adsorption onto quartz filters in the air sampling. The small variations ensures that the two main classes of constituents (OC and EC) were both efficiently extracted.

#### Reference particles

Carbon black Printex90 was provided by Evonik Degussa GmbH (Frankfurt, Germany) [5, 73, 83]. Diesel particle SRM 2975 (referred to as NIST2975) was obtained from the National Institute of Standards and Technology (Gaithersburg, MD, USA). The certificate of analysis is avaiable at http://www.nist.gov.

### Transmission electron microscopy (TEM) and analysis of organic (OC) and elemental (EC) carbon

The organic carbon (OC) and elemental carbon (EC) of the extracted particles were measured with a thermal-optical carbon analyzer (Sunset Laboratory Inc.), using the EUSAAR_2 protocol. To analyze the soot particles microstructure, samples were collected with electrostatic precipitation (Nanometer Aerosol Sampler, model 3089, TSI Inc.) on Cu-grids with lacey carbon coating (Whatman PTFE, 150 mm, pore size 5 μm) as well as the extracted particles were deposited in instillation vehicle on Cu-grids with lacey carbon coating (data not shown) and imaged by transmission electron microscopy (JEOL 3000F) operating at 300 kV. The primary particle determination and estimation of specific surface area (SSA) is described in Gren et al. [31]. In brief, the primary particle diameter was manually measured with ImageJ software [84]. The specific surface area (SSA) of each primary particle was estimated by using the primary particle size (d_pp) and diesel soot density (ρ_pp) of 1.8 μg/m^3^ [32] with the formula SSA =6/(ρ_pp · d_pp) [85]. By assuming point contact between the primary particles in the agglomerates, the geometric mean of the lognormal distribution of SSA of all measured primary particles within a sample was used as the mean SSA. The particle mobility size distribution in the diluted exhaust was measured from the dilution tunnel with a fast particulate analyzer (model DMS500, Cambustion Ltd.). The fast particulate analyzer measures the number size distribution of 5–1000 nm by classification of particles by their electrical mobility.

### PAHs and metal analysis

#### PAHs

Particle samples, blank control filters and standard reference material NIST2975 were solvent-extracted using dichloromethane, as previously described in detail [31]. Extracts were analyzed for 20 native PAHs, 13 alkylated PAHs, 14 nitrated PAHs, 10 oxygenated PAHs and 6 dibenzothiophenes (DBT), using an Agilent 5975C mass spectrometer (MS) coupled to a 7890A gas chromatograph (GC, Agilent Technologies). Names, nominal masses, retention times and the associated deuterium labeled internal standards (IS) and recovery standards (RS) for all investigated compounds are shown in Additional file K.

Only one PAH analysis was performed for each combustion condition, however, at least two separate filter collections were pooled to account for engine variability.

#### Metals

The metal analysis was carried out as previously described in Bendtsen et al. [12], but with slightly modified extraction times. Briefly, as it was not possible to transfer the amount of ≤1 mg particle matter from the received vials to containers suitable for microwave-assisted acid digestion, a volume of 1 mL of 25% (v/v) nitric acid was directly added to the vials. For the preparation of reference material NIST2975 and CB (*n* = 2), 1 mg of material were weighed into 13 mL polypropylene tubes (Sarstedt, Nümbrecht, Germany) and 1 mL of 25% (v/v) nitric acid added. Samples were first agitated at 600 oscillations per min overnight (Stuart Scientific SF1 shaker), then incubated for approximately 7 h at room temperature without agitation and then shaken for another 72 h and transferred with 6 mL of ultrapure water into polypropylene tubes. Before analysis, the samples were centrifuged for 5 min at 4500 x g (Heraeus Multifuge X3 FR, Thermo Scientific), because no complete digestion of the particles was achieved. A volume of 5 mL of the supernatant was transferred to a new polypropylene tube and diluted 5-fold with 5% nitric acid. A triple quadrupole inductive coupled plasma mass spectrometer (ICP-MS) (Agilent 8900 ICP-QQQ, Santa Clara, USA) equipped with a MicroMist borosilicate glass concentric nebulizer and a Scott type double-pass water-cooled spray chamber was run in no gas (Cd, Hg, Pb, Bi, U) or helium (remaining elements) mode with 0.1–3 s integration time per mass. Quantification was performed based on external calibration.

##### Reactive oxygen species (ROS) assay

The level of ROS generated by the DEPs and CB were determined using the acellular 2′,7′ dichlorodihydrofluorescein diacetate (DCFH_2_-DA) assay. We used the protocol previously described in detail [77, 86] with the addition, that all materials were tested for auto-fluorescence as this may interfere with the assay. CB Printex 90 was tested alongside the DEPs as benchmark particle. Briefly, the DCFH_2_-DA (#D399, Invitrogen) was chemically hydrolyzed in the dark with NaOH to generate 2′,7′ dichlorodihydrofluorescein (DCFH_2_), which was further diluted with phosphate buffer (pH 7.4) to 0.04 mM. The PM suspensions were prepared using 16 min sonication (Branson S-450D) in Hank’s balanced saline solution (HBSS, without phenol, #H6648, Sigma Aldrich). The PM suspensions were further diluted in HBSS and tested at 0 μg/ml and eight doubling PM concentrations from 1.05 up to 101.25 μg/ml. The final concentration of DCFH_2_ at assay start was 0.01 mM. Generated ROS caused formation of 2′,7′ dichlorofluorescein (DCF) from DCFH_2_ that was spectrofluorimetrically measured following 3 h of incubation in the dark (37 °C and 5% CO_2_). Excitation and emission wavelengths were λex = 490 nm and λem = 520 nm, respectively (Victor Wallac-21,420; PerkinElmer, Skovlunde, Denmark). Auto-fluorescence was measured by replacing the ROS probe by Hank’s balanced saline solution (HBSS) at the highest tested PM concentrations.

##### Dynamic light scattering

Dynamic Light Scattering (DLS) was used to analyze hydrodynamic size distributions of particles in suspension (Malvern Zetasizer Nano ZS, Malvern Instruments Ltd., UK). Determinations were carried out directly in the solutions used for instillation in 1 ml polystyrene cuvettes at 25 °C. Six repeated same sample measurements were analyzed and average was calculated. For the calculation of hydrodynamic size, values from reference particle carbon black Printex90 was used (refractive (Ri) = 2.020, absorption indices (Rs) = 2000) for all particles, with standard optical and viscosity properties for H_2_O.

### Mice

The study was in agreement with Directive 2010/63/EU of the European Parliament and of the Council of 22 September 2010 on the protection of mice used for scientific purposes, and the Danish Animal Experimentation Act (LBK 474 15/05/2014). The study was approved by The Animal Experiments Inspectorate under The Ministry of Environment and Food of Denmark (License: 2015-15-0201-00465) and the local Animal Welfare Committee responsible for ensuring implementation of 3R policy at the National Research Center for the Working Environment.

For this study, 492 female C57BL/6Tac mice were used. They were 7 weeks old at arrival and group-housed in standard cages with 6–8 mice with ad libitum access to tap water and Altromin 1324 rodent diet. All mice were housed in 1290D euro standard Type 3 cages on saw dust bedding with mouse house, wooden chew blocks and Enviro Dri nesting material as enrichment. The mice were kept at 21 ± 1 °C and 50 ± 10% humidity in a 12 h light-dark circle.

#### Study design

After one week of acclimatization, mice were exposed to a single dose of collected particles of either 6 μg, 18 μg or 54 μg per mouse by intratracheal instillation (6–8 mice per dose per exposure).

As it is only possible to expose a certain number of mice per day, the study was spread out on several days. First cohort was exposed to RME13 and CB, second cohort was exposed to DEP13, third cohort was exposed to DEP9.7, fourth cohort was exposed to DEP17, and fifth cohort was exposed to HVO13. For each exposure cohort there were four vehicle control mice, which were pooled together as one final control group for each post-exposure day for data evaluation. All cohorts were euthanized on day 1, day 28 and day 90 post-exposure. On day 28 and day 90, five additional mice per exposure were dedicated for histology (except for CB). In groups subjected to 1-day exposure to DEP13, DEP9.7, DEP17 and HVO13, some animals were initially not correctly dosed or cell recovery procedures failed upon euthanization. Therefore, a new cohort of mice was exposed, comprising two animals per dose for 1-day exposures, supplementing the groups from the original study.

#### Instillation procedure

Particles were suspended in Nanopure Diamond Water with 0.1% Tween80 and sonicated for 16 min using a Branson Sonifier S-450D (Branson Ultrasonics Corp, Danbury, CT, USA) (see description in [62]). The suspensions were diluted and re-sonicated for 2 min. Nanopure Diamond Water with 0.1% Tween80 was prepared similarly as vehicle. All solutions were prepared fresh for each instillation day and instilled within 1 h.

Instillation procedure was carried out as previously described [12, 87]. In brief, mice were exposed during isoflurane anesthesia to the suspended particles in vertical position with back support. A diode light was placed at the larynx visualizing the breathing pattern, to ensure correct delivery. 200 μl air was placed in the syringe after the instillation volume (50 μl) and administered post-exposure, to ensure maximum delivery into the lung. The mice were then returned to the home cage, placed on a heating plate, to ensure optimal recovery from anesthesia. Following the procedure and until euthanization, the mice were under observation for signs of discomfort. In case of weight loss (maximum 20%) and/or clear signs of discomfort (ruffled fur, isolation, facial pain expression, changed respiration, reduced activity), the mouse was taken out of the study and euthanized.

#### Organ harvest and preparation

Sedation, bronchoalveolar lavage (BAL), euthanization, organ harvest and procedures for analysis of mRNA, protein, and DNA strand breaks in the Comet assay were carried out as previously described [12].

##### Statistical analysis of in vivo data

Data was analyzed with GraphPad Prism (GraphPad Prism, version 7.03 for Windows, GraphPad Software, La Jolla California USA, www.graphpad.com). BAL fluid data was log10 transformed to achieve normal distribution. If no cells were counted across all cell types for one mouse, for instance due to high number of erythrocytes, it was considered as a cell processing error and the mouse was removed from the particular dataset. Values of zero within one cell type were replaced by the value of 0.25, generating an arbitrary low value of < 500 cells, considered as the detection limit of the differential count. Parametric data were analyzed by one-way ANOVA followed by Dunnett’s (comparison to control group). Nonparametric data was analyzed by Kruskal-Wallis followed by Dunn’s multiple comparisons test. Linear regressions were performed on log-transformed data, after testing the normality of the residuals.

## Supplementary information

**Additional file 1.**

## Data Availability

The datasets used and/or analyzed during the current study are available from the corresponding author on reasonable request.
