# Supplementary material for: Particle characterization and toxicity in C57BL/6 mice following instillation of five different diesel exhaust particles designed to differ in physicochemical properties
Source: Part Fibre Toxicol. 2020 Aug 8;17:38. doi: 10.1186/s12989-020-00369-9 (PMC7414762; doi:10.1186/s12989-020-00369-9)
Supplement: Supplementary file 1 — Additional file 1. [file 12989_2020_369_MOESM1_ESM.docx]

**Additional File**

**A. Detailed results of PAH analysis**

Table

| **Table**. **Measured content of PAHs and PAH derivatives in diesel emission particles and NIST2975 reference material (µg/g particles).** | | | | | | |
| --- | --- | --- | --- | --- | --- | --- |
|  | **DEP** | | | **HVO** | **RME** | *Reference* |
|  | MK1 low-sulfur diesel | | | Hydrotreated vegetable oil | Rapeseed methyl ester | NIST2975* |
| **O_2_ %** | **10** | **13** | **17** | **13** | **13** |  |
| **Native PAH** | | | | | | |
| naphthalene | 4.4 | 136.8 | 6.8 | 52.8 | 19.3 | 1.4 [1.8] |
| biphenyl | 5.0 | 67.7 | 2.7 | 63.2 | 3.5 | 0.3 [0.3] |
| acenaphthylene | 16.2 | 96.9 | 2.8 | 68.7 | 5.7 | <LOD |
| acenaphthene | 1.0 | 17.0 | 2.0 | 3.5 | 0.5 | 0.1 |
| fluorene | 17.5 | 44.8 | 2.9 | 62.5 | 5.1 | 0.5 [0.4] |
| phenanthrene | 423.7 | 236.9 | 44.7 | 1167.4 | 207.8 | 10.6 [17.3] |
| anthracene | 60.9 | 17.8 | 3.3 | 47.9 | 21.4 | 0.1 [0.0] |
| fluoranthene | 1677.0 | 141.4 | 162.2 | 1591.4 | 325.2 | 23.3 [26.9] |
| pyrene | 2381.6 | 182.8 | 223.2 | 2341.4 | 293.1 | 0.8 [0.9] |
| retene | 9.1 | 271.8 | 166.1 | 120.6 | 157.6 | <LOD |
| benzo(a)anthracene | 1378.15 | 5.5 | 15.2 | 187.7 | 6.3 | 0.2 [0.3] |
| chrysene | 2609.2 | 9.3 | 52.8 | 369.9 | 15.8 | 3.9 [4.6] |
| benzo(b)fluoranthene | 4158.16 | 207.6 | 40.7 | 548.4 | 27.8 | 8.2 [11.5] |
| benzo(k)fluoranthene | 1094. | 887.6 | 59.0 | 187.9 | 36.2 | 0.3 [0.7] |
| benzo(a)pyrene | 3739.3 | 61.3 | 32.8 | 759.2 | 33.4 | 0.3 [0.0] |
| perylene | 638.3 | 14.5 | 10.0 | 137.6 | 2.5 | 0.1 [0.0] |
| indeno(1,2,3-c,d)pyrene | 2072.1 | 2.3 | 8.7 | 702.5 | 2.1 | 0.9 [1.4] |
| dibenzo(a,h)anthracene | 40.2 | 27.2 | 7.1 | 49.9 | 4.3 | 0.4 [0.3] |
| benzo(g,h,i)perylene | 2608.5 | 31.6 | 14.3 | 1216.6 | 7.6 | 0.9 [0.5] |
| coronene | 752.3 | 7.6 | 0.5 | 280.5 | 0.8 | 0.3 |
| **SUM Native PAH** | 23686.3 | 2468.7 | 857.6 | 9959.3 | 1176.1 | 52.4 |
| **Alkyl-PAH** | | | | | | |
| 2-methylnaphthalene | <LOD | 150.3 | 2.8 | 58.3 | 10.4 | 0.8 [1.9] |
| 1-methylnaphthalene | 1.4 | 106.5 | 2.5 | 30.8 | 6.4 | 0.4 [1.0] |
| 2,3-dimethylnaphthalene | 3.8 | 51.8 | 7.3 | 14.6 | 13.5 | 2.4 |
| 2,3,5-trimethylnaphthalene | 0.2 | 14.7 | 2.1 | 3.3 | 0.8 | 0.2 |
| 1-methylfluorene | 0.4 | 11.1 | 3.3 | 7.8 | 3.3 | 0.1 |
| 4-methylphenanthrene | 8.5 | 16.9 | 7.1 | 15.5 | 9.7 | 0.6 |
| 3-methylphenanthrene | 11.0 | 14.1 | 7.8 | 18.6 | 13.3 | 1.2 [1.0] |
| 1-methylphenanthrene | 11.5 | 16.6 | <LOD | 18.1 | 56.0 | <LOD |
| 1-methylanthracene | 17.6 | 16.6 | 6.9 | 23.3 | 11.9 | 0.6 |
| 2-phenylnaphthalene | 83.1 | 25.5 | 6.3 | 81.1 | 26.9 | 1.1 |
| 1-methylfluoranthene | 41.8 | 23.7 | 6.9 | 98.6 | 17.2 | 0.2 |
| 1-methylpyrene | 166.8 | 34.7 | 23.3 | 268.3 | 30.4 | 0.0 |
| 2-methylchrysene | 54.4 | 0.4 | 1.2 | 6.1 | 0.7 | 0.0 |
| **SUM Alkyl-PAH** | 400.4 | 482.9 | 77.2 | 644.3 | 150.5 | 7.5 |
| **Table (cont.) Measured content of PAHs and PAH derivatives in diesel emission particles and NIST2975 reference material (µg/g particles).** | | | | | | |
| **DBT (Dibenzothiophenes)** | | | | | | |
| dibenzothiophene | 44.1 | 52.3 | 99.7 | 61.1 | 72.0 | 10.3 |
| 2-methyldibenzothiophene | 1.1 | 4.8 | 13.1 | 8.0 | 6.4 | 0.1 |
| 1-methyldibenzothiophene | 0.4 | 12.4 | 6.6 | 10.6 | 8.4 | 0.2 |
| 4-methyldibenzothiophene | 0.2 | 1.0 | 1.2 | 0.9 | 0.7 | 0.0 |
| 2,8-dimethyldibenzothiophene | 0.5 | 5.9 | 4.0 | 4.1 | 4.5 | 0.0 |
| 2,4,7-trimethyldibenzothiophene | 0.5 | 1.9 | 3.3 | 1.8 | 2.5 | <LOD |
| **SUM DBT** | 46.8 | 78.2 | 127.8 | 86.3 | 94.5 | 10.5 |
| **Nitro-PAH** | | | | | | |
| 1-Nitronaphthalene | 0.1 | 1.6 | 0.1 | 2.2 | 3.0 | 0.0 [0.0] |
| 2-Nitronapthalene | 0.3 | 11.9 | 0.8 | 22.7 | 13.0 | 0.1 [0.1] |
| 5-nitro acenapthalene | 1.3 | <LOD | 2.0 | 0.5 | <LOD | <LOD |
| 2-Nitrofluorene | 0.1 | 0.0 | 0.0 | 0.1 | 0.5 | 0.1 |
| 9-Nitroanthracene | 0.6 | 0.0 | 0.0 | 0.1 | <LOD | 1.6 [3.0] |
| 9-Nitrophenanthrene | 0.7 | 0.0 | 0.0 | 0.2 | 0.0 | 0.2 |
| 4-Nitropyrene | 9.4 | 0.0 | 0.3 | 3.5 | 1.6 | 0.1 [0.2] |
| 3-Nitrofluoranthene | 9.2 | 0.0 | <LOD | 1.2 | 2.1 | 2.2 |
| 1-Nitropyrene | 2.4 | 0.1 | 4.0 | 3.1 | 6.5 | 25.0 |
| 2-Nitropyrene | 88.6 | 0.2 | 0.2 | 26.2 | 1.8 | 0.0 |
| 7-Nitrobenz[a]anthracene | 7.0 | 6.4 | <LOD | 3.7 | 9.4 | 3.2 |
| 6-Nitrochrysene | 0.2 | <LOD | <LOD | 0.0 | 1.7 | 0.8 |
| 3-Nitrobenzanthrone | 0.3 | 0.1 | 0.2 | 0.7 | 0.3 | 0.2 |
| 6-Nitrobenzo[a]pyrene | 10.4 | 0.8 | 0.8 | 1.3 | 0.5 | 0.2 [1.4] |
| **SUM Nitro-PAHs** | 130.5 | 21.2 | 8.4 | 65.5 | 40.4 | 33.7 |
| **Oxy-PAH** | | | | | | |
| Napthalene-1-aldehyde | 17.7 | 246.9 | 11.9 | 259.9 | 27.5 | 3.0 |
| 2-Naphthaldehyde | 67.6 | 1005.5 | 104.0 | 947.4 | 248.0 | 2.8 |
| p-Fluorenone | 136.0 | 168.3 | 49.7 | 659.8 | 214.5 | 12.7 |
| 9,10 Anthraquinone | 146.6 | 10.5 | 31.9 | 184.8 | 72.6 | 18.0 |
| 1,4 Anthraquinone | 25.2 | 6.5 | 1.3 | 2.7 | <LOD | <LOD |
| Phenanthrene-9-aldehyde | 50.3 | 0.5 | 2.8 | 21.7 | 0.1 | 2.0 |
| Benzanthrone | 1475.6 | 1.3 | 21.6 | 425.3 | 19.2 | 10.3 |
| Benz[a]anthracene-7,12-dione | 201.3 | 0.1 | 0.8 | 8.0 | 2.7 | 13.2 |
| **SUM Oxy-PAH** | 2488.3 | 1452.3 | 314.3 | 2627.5 | 596.3 | 265.1 |
| *For comparison, in [] PAH levels quantified after soxhlet extraction or PFE at 100-200 °C, as reported by NIST.  The certificate of analysis is available at <http://www.nist.gov>. | | | | | | |

**B. Dynamic Light Scattering**

Figure and Table


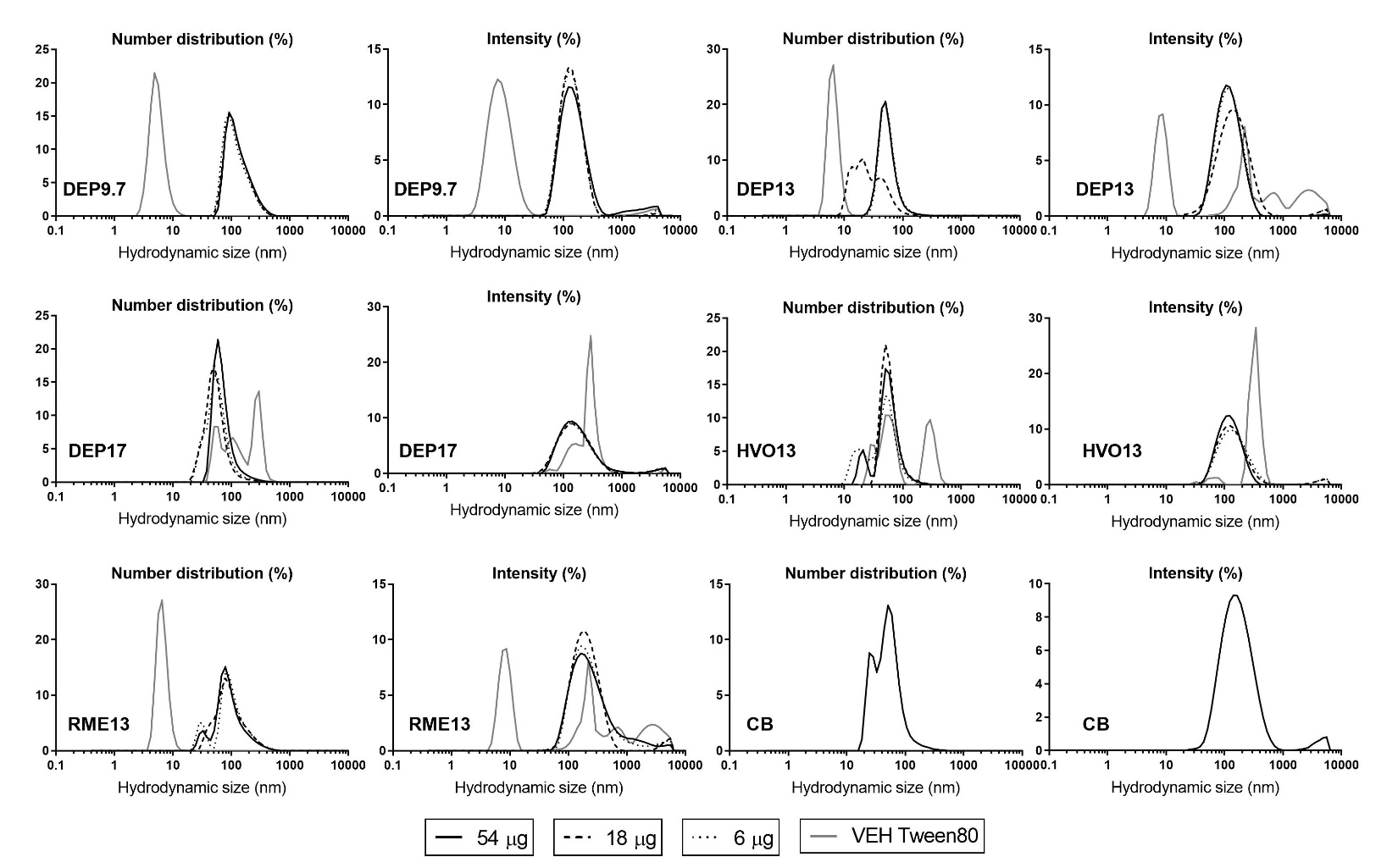


| **Size distribution in dispersion for five generated diesel particles and CB** | | | | | | |
| --- | --- | --- | --- | --- | --- | --- |
| **Dose** | ***6 µg*** | | ***18 µg*** | | ***54 µg*** | |
|  | *Z_ave_ (d.nm)* | *PdI* | *Z_ave_ (d.nm)* | *PdI* | *Z_ave_ (d.nm)* | *PdI* |
| DEP9.7 | 167.95 | 0.191 | 168.6833 | 0.159833 | 189.05 | 0.238333 |
| DEP13 | 108.5167 | 0.234667 | 122.0167 | 0.270667 | 103.9833 | 0.195167 |
| DEP17 | 140.95 | 0.3161667 | 140.9 | 0.2986667 | 148.11667 | 0.2755 |
| HVO13 | 126.25 | 0.2968333 | 120.16667 | 0.2651667 | 109.33333 | 0.1813333 |
| RME13 | 199.55 | 0.31 | 182.06667 | 0.2505 | 196.86667 | 0.3081667 |
| CB |  |  |  |  | 145.4833 | 0.264667 |
| Vehicle* | Z-average: 487.2011 (10.552-1263.767). PdI: 0.6292 (0.2055-0.91) | | | | | |
| DEP: MK1 low-sulfur diesel at 9.7, 13, and 17% intake O_2_ concentration, HVO: Hydrotreated vegetable oil at 13% intake O_2_ concentration, RME: Rapeseed methyl ester at 13% intake O_2_ concentration. CB: Carbon black Printex90.  All particles were dispersed in Nanopure water with 0.1 % Tween80 (Vehicle). Z-Average (intensity based harmonic mean) relates to particle sizes and Polydispersity Index (PdI) relates to the distribution. Values are mean of six consecutive measurements. *Mean and min/max of means of vehicle samples measured along with the different particle dispersions. | | | | | | |

**C. Cell composition in BAL fluid**

Table

| **Table. BAL fluid cell composition on day 1, 28 and 90 post-exposure.** | | | | | | | | | | | |
| --- | --- | --- | --- | --- | --- | --- | --- | --- | --- | --- | --- |
| **Day 1** | | **Total cell count** | | **Neutrophils** | | **Macrophages** | | **Eosinophils** | | **Lymphocytes** | |
|  |  | Mean | SD | Mean | SD | Mean | SD | Mean | SD | Mean | SD |
| *Vehicle* | | 49083 | 21977 | 4392 | 5597 | 40993 | 16788 | 590 | 1734 | 457 | 539 |
| *Blank filter extraction* | | 39573 | 15841 | 2202 | 2659 | 34399 | 13836 | 15,8 | 38,8 | 481 | 448 |
| *CB 54 µg* | | 55117 | 7831 | 11166 | 6892 | 41430 | 11522 | 824 | 1139 | 565 | 655 |
| DEP9.7 | *6 µg* | 47617 | 18185 | 2699 | 3033 | 40864 | 15650 | 1375 | 1793 | 258 | 391 |
|  | *18 µg* | 49286 | 35048 | 5055 | 8800 | 41326 | 25719 | 401 | 525 | 130 | 188 |
|  | *54 µg* | 141863 | 199234 | 92629 | 182949 | 40766 | 21942 | 5849 | 14159 | 619 | 1036 |
| DEP13 | *6 µg* | 43567 | 15624 | 1757 | 1387 | 36608 | 12268 | 2896 | 4362 | 815 | 865 |
|  | *18 µg* | 44067 | 14117 | 5979 | 7024 | 34286 | 7724 | 1277 | 2001 | 300 | 428 |
|  | *54 µg* | 134417 | 117048 | 60401 | 68500 | 49638 | 29130 | 18683 | 34566 | 2859 | 3520 |
| DEP17 | *6 µg* | 49650 | 16438 | 902 | 1004 | 45019 | 15721 | 183 | 237 | 191 | 227 |
|  | *18 µg* | 45357 | 8936 | 1211 | 958,2 | 40328 | 8546 | 580 | 1470 | 842 | 1712 |
|  | *54 µg* | 106867 | 50884 | 56841 | 45747 | 43391 | 17119 | 3799 | 7769 | 1046 | 495 |
| HVO13 | *6 µg* | 53114 | 33757 | 4396 | 4224 | 45291 | 31914 | 569 | 604 | 420 | 473 |
|  | *18 µg* | 44800 | 21610 | 5313 | 4443 | 34586 | 16624 | 1538 | 1740 | 496 | 556 |
|  | *54 µg* | 104850 | 53279 | 65285 | 37707 | 29295 | 13740 | 5352 | 5536 | 1796 | 1754 |
| RME13 | *6 µg* | 47850 | 18454 | 5820 | 2020 | 39032 | 17310 | 205 | 183 | 604 | 221 |
|  | *18 µg* | 56983 | 8517 | 4238 | 1996 | 46882 | 9326 | 3082 | 4067 | 1199 | 532 |
|  | *54 µg* | 56760 | 26039 | 10043 | 4613 | 42352 | 22367 | 2190 | 2260 | 397 | 440 |
| **Day 28** | | **Total cell count** | | **Neutrophils** | | **Macrophages** | | **Eosinophils** | | **Lymphocytes** | |
|  |  | Mean | SD | Mean | SD | Mean | SD | Mean | SD | Mean | SD |
| *Vehicle* | | 49655 | 16818 | 3084 | 4720 | 40359 | 13334 | 3539 | 9013 | 1703 | 4835 |
| *Blank filter extraction* | | 40383 | 11545 | 1240 | 734 | 35904 | 10457 | 1369 | 2588 | 441 | 438 |
| *CB 54 µg* | | 77817 | 49998 | 10732 | 7467 | 51563 | 24084 | 61,8 | 151 | 12220 | 20680 |
| DEP9.7 | *6 µg* | 61033 | 19356 | 2111 | 1300 | 55392 | 17629 | 251 | 616 | 469 | 277 |
|  | *18 µg* | 47617 | 23156 | 741 | 619 | 43471 | 20872 | 501 | 1228 | 345 | 355 |
|  | *54 µg* | 43717 | 7944 | 405 | 399 | 41184 | 7444 | 273 | 426 | 237 | 288 |
| DEP13 | *6 µg* | 66633 | 30364 | 15170 | 14107 | 38570 | 7566 | 7577 | 15428 | 1244 | 1764 |
|  | *18 µg* | 39317 | 15662 | 144 | 227 | 36798 | 14794 | 75,2 | 184 | 150 | 273 |
|  | *54 µg* | 38458 | 25771 | 1975 | 2016 | 32835 | 21291 | 412 | 707 | 1364 | 1197 |
| DEP17 | *6 µg* | 47833 | 10981 | 1633 | 1699 | 42118 | 11002 | 82 | 130 | 526 | 370 |
|  | *18 µg* | 55183 | 13391 | 9163 | 10540 | 41697 | 3635 | 445 | 665 | 771 | 563 |
|  | *54 µg* | 50417 | 16032 | 808 | 437 | 45095 | 13164 | 579 | 1311 | 815 | 1225 |
| HVO13 | *6 µg* | 37617 | 9481 | 1699 | 1310 | 32995 | 7805 | 499 | 609 | 466 | 467 |
|  | *18 µg* | 47483 | 22775 | 966 | 787 | 42646 | 21357 | 73,3 | 179 | 858 | 1101 |
|  | *54 µg* | 37960 | 9932 | 808 | 503 | 33757 | 9560 | 542 | 1125 | 470 | 509 |
| RME13 | *6 µg* | 61383 | 22202 | 12304 | 8539 | 44743 | 26032 | 1738 | 3228 | 514 | 404 |
|  | *18 µg* | 50400 | 17604 | 9765 | 5528 | 36208 | 15339 | 1389 | 3271 | 657 | 1005 |
|  | *54 µg* | 50533 | 12635 | 4231 | 1368 | 42009 | 9594 | 1286 | 3151 | 903 | 1408 |

| **Table (cont.) BAL fluid cell composition on day 1, 28 and 90 post-exposure.** | | | | | | | | | | | |
| --- | --- | --- | --- | --- | --- | --- | --- | --- | --- | --- | --- |
| **Day 90** | | **Total cell count** | | **Neutrophils** | | **Macrophages** | | **Eosinophils** | | **Lymphocytes** | |
|  |  | Mean | SD | Mean | SD | Mean | SD | Mean | SD | Mean | SD |
| *Vehicle* | | 47390 | 18085 | 430 | 525 | 42254 | 17041 | 489 | 770 | 658 | 607 |
| *Blank filter extraction* | | 45020 | 17583 | 536 | 850 | 40727 | 17284 | 471 | 881 | 799 | 958 |
| DEP9.7 | *54 µg* | 55040 | 6582 | 967 | 470 | 50738 | 5976 | 0 | 0 | 320 | 224 |
| DEP13 | *54 µg* | 65067 | 17587 | 3253 | 1554 | 52616 | 15273 | 1001 | 1553 | 3698 | 2042 |
| DEP17 | *54 µg* | 52183 | 14315 | 1249 | 900 | 46581 | 14126 | 749 | 1651 | 1047 | 1359 |
| HVO13 | *54 µg* | 39350 | 7460 | 443 | 766 | 30271 | 15140 | 3960 | 8855 | 617 | 1096 |
| RME13 | *54 µg* | 47850 | 22499 | 460 | 422 | 41911 | 18337 | 1119 | 1566 | 850 | 895 |
| DEP: MK1 low-sulfur diesel at 9.7, 13, and 17% intake O2 concentration, HVO: Hydrotreated vegetable oil at 13% intake O2 concentration, RME: Rapeseed methyl ester at 13% intake O2 concentration. CB: Carbon black Printex90. CB was not evaluated on day 90.  **Number of animals per group** Day 1: VEH=24, Blank filter=6, Particles=6-8. Day 28: Total cells; VEH=20, others=6; Neutr., Macr., Eosino., Lymph.: VEH=20-28, Particles=5-6. Day 90: Total cells; VEH=10, others=4-6; Neutr., Macr., Eosino., Lymph.: VEH=10-14, others=4-6 | | | | | | | | | | | |
|  |  |  |  |  |  |  |  |  |  |  |  |

**D. Cell composition in BAL fluid on day 28**

Figure

**
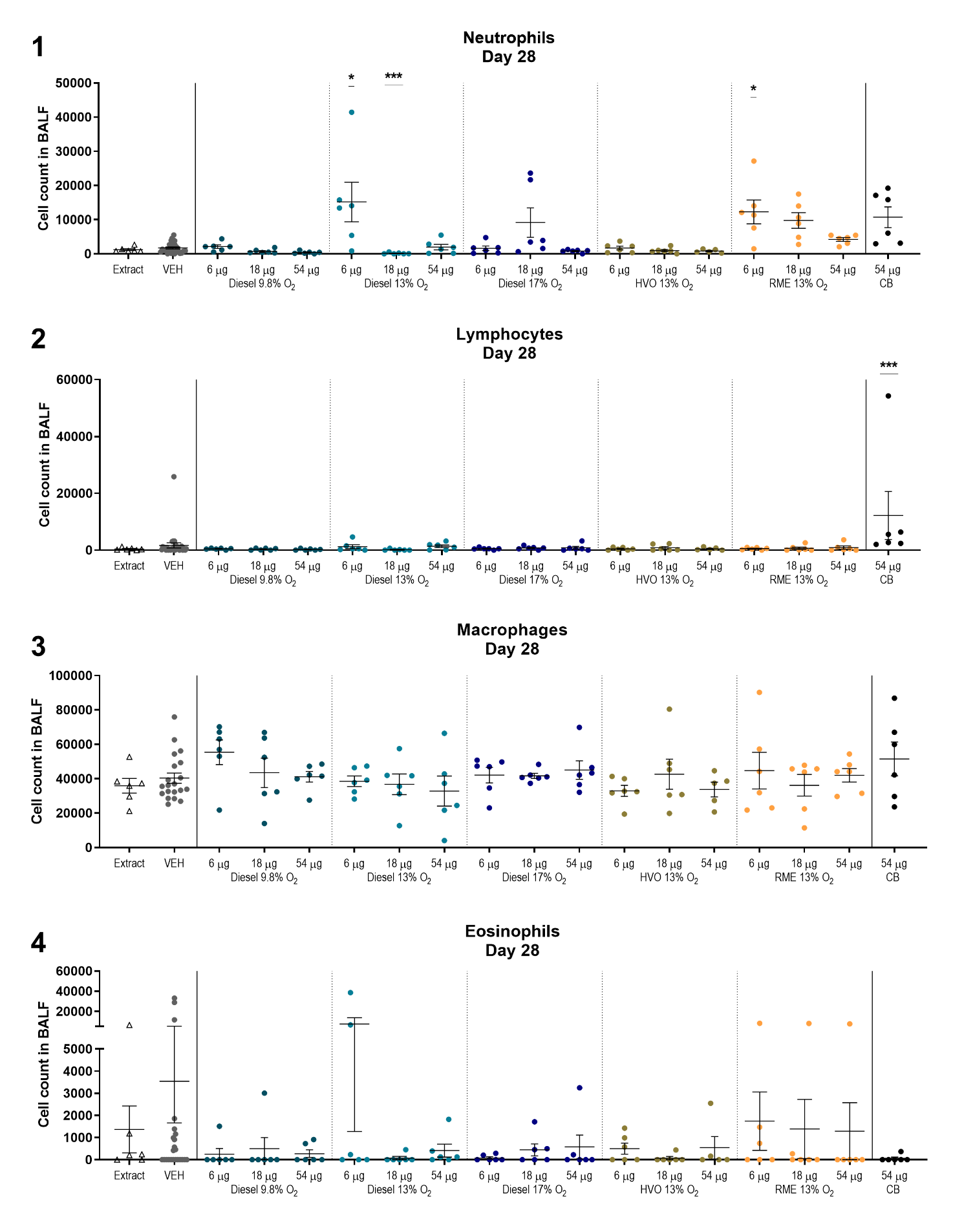
**

Diesel: MK1 low-sulfur diesel at 9.7, 13, and 17% intake O2 concentration, HVO: Hydrotreated vegetable oil at 13% intake O2 concentration, RME: Rapeseed methyl ester at 13% intake O2 concentration. CB: Carbon black Printex90. Cell counts of bronchoalveolar lavage of mice post-exposure to 6, 18, and 54 µg particles. 1. Neutrophils. 2. Lymphocytes. 3. Macrophages 4. Eosinophils. * = p<0.05. ** = p<0.01, *** = p<0.001, **** = p<0.0001.

**E. Neutrophil influx day 1 and *Saa3* mRNA in lung day 1**

Figure

**
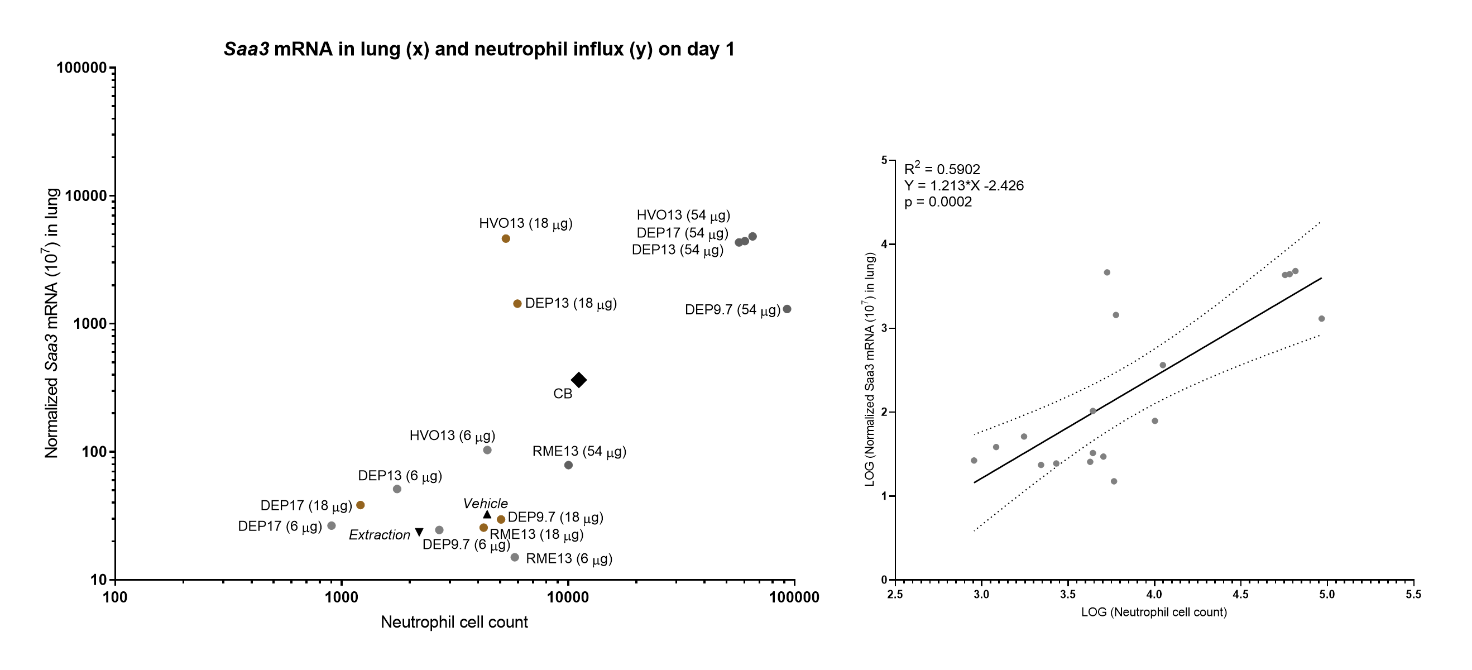
**

Neutrophil influx correlated with Saa3 mRNA levels in lung on day 1. Original data (left panel) and linear regression plot (right panel).

**F. DNA strand breaks**

Table

| **Table. Tail length in Comet assay on day 1, 28 and 90 post-exposure.** | | | | | | | |
| --- | --- | --- | --- | --- | --- | --- | --- |
| Day 1 | | BAL | | LUNG | | LIVER | |
| **Exposure** | *Dose* | Mean and SD | | Mean and SD | | Mean and SD | |
| ***Extract*** | *0* | 14 | 2 | 20 | 4 | 14 | 3 |
| ***VEH*** | *0* | 15 | 3 | 23 | 3 | 16 | 3 |
| ***DEP9.7*** | *6* | 13 | 1 | 24 | 8 | 14 | 1 |
|  | *18* | 12 | 2 | 20 | 3 | 14 | 2 |
|  | *54* | 14 | 2 | 20 | 3 | 14 | 2 |
| ***DEP13*** | *6* | 13 | 3 | 19 | 4 | 14 | 3 |
|  | *18* | 14 | 4 | 21 | 2 | 16 | 3 |
|  | *54* | 13 | 3 | 22 | 3 | 19 | 8 |
| ***DEP17*** | *6* | 15 | 4 | 22 | 4 | 14 | 1 |
|  | *18* | 14 | 1 | 17 | 2 | 14 | 3 |
|  | *54* | 14 | 3 | 17 | 1 | 15 | 3 |
| ***HVO13*** | *6* | 12 | 4 | 19 | 4 | 14 | 2 |
|  | *18* | 14 | 3 | 18 | 5 | 14 | 2 |
|  | *54* | 14 | 3 | 23 | 6 | 13 | 1 |
| ***RME13*** | *6* | 16 | 5 | 21 | 2 | 15 | 1 |
|  | *18* | 14 | 2 | 21 | 4 | 16 | 2 |
|  | *54* | 17 | 2 | 21 | 3 | 17 | 1 |
| ***CB*** | *54* | 15 | 1 | 20 | 2 | 15 | 2 |
| Day 28 | | BAL | | LUNG | | LIVER | |
| **Exposure** | *Dose* | Mean and SD | | Mean and SD | | Mean and SD | |
| ***Extract*** | *0* | 13 | 1 | 19 | 7 | 13 | 3 |
| ***VEH*** | *0* | 17 | 4 | 22 | 6 | 23 | 7 |

| **Table (cont.). Tail length in Comet assay on day 1, 28 and 90 post-exposure.** | | | | | | | |
| --- | --- | --- | --- | --- | --- | --- | --- |
| Day 28 | | BAL | | LUNG | | LIVER | |
| **Exposure** | *Dose* | Mean and SD | | Mean and SD | | Mean and SD | |
| ***DEP9.7*** | *6* | 13 | 3 | 15 | 2 | 17 | 2 |
|  | *18* | 15 | 1 | 16 | 3 | 18 | 5 |
|  | *54* | 16 | 2 | 14 | 2 | 17 | 4 |
| ***DEP13*** | *6* | 19 | 3 | 18 | 5 | 21 | 6 |
|  | *18* | 17 | 3 | 18 | 3 | 21 | 5 |
|  | *54* | 16 | 1 | 18 | 3 | 18 | 3 |
| ***DEP17*** | *6* | 15 | 1 | 19 | 5 | 17 | 2 |
|  | *18* | 13 | 1 | 14 | 2 | 19 | 3 |
|  | *54* | 13 | 2 | 24 | 2 | 26 | 3 |
| ***HVO13*** | *6* | 13 | 3 | 23 | 5 | 22 | 3 |
|  | *18* | 13 | 2 | 21 | 5 | 20 | 3 |
|  | *54* | 12 | 2 | 21 | 5 | 17 | 4 |
| ***RME13*** | *6* | 16 | 4 | 20 | 5 | 26 | 2 |
|  | *18* | 17 | 3 | 23 | 3 | 24 | 2 |
|  | *54* | 14 | 1 | 22 | 4 | 22 | 3 |
| ***CB*** | *54* | 13 | 4 | 21 | 8 | 21 | 2 |
| Day 90 | | BAL | | LUNG | | LIVER | |
| **Exposure** | *Dose* | Mean and SD | | Mean and SD | | Mean and SD | |
| ***Extract*** | *0* | 11 | 0 | 14 | 3 | 14 | 2 |
| ***VEH*** | *0* | 14 | 3 | 24 | 5 | 23 | 7 |
| ***DEP9.7*** |  |  |  |  |  |  |  |
|  |  |  |  |  |  |  |  |
|  | *54* | 13 | 2 | 16 | 1 | 14 | 2 |
| ***DEP13*** |  |  |  |  |  |  |  |
|  |  |  |  |  |  |  |  |
|  | *54* | 14 | 2 | 17 | 2 | 17 | 2 |
| ***DEP17*** |  |  |  |  |  |  |  |
|  |  |  |  |  |  |  |  |
|  | *54* | 13 | 1 | 24 | 3 | 23 | 2 |
| ***HVO13*** |  |  |  |  |  |  |  |
|  |  |  |  |  |  |  |  |
|  | *54* | 12 | 2 | 21 | 4 | 23 | 4 |
| ***RME13*** |  |  |  |  |  |  |  |
|  |  |  |  |  |  |  |  |
|  | *54* | 13 | 2 | 17 | 7 | 20 | 3 |
| ***CB*** |  |  |  |  |  |  |  |
| DEP: MK1 low-sulfur diesel at 9.7, 13, and 17% intake O2 concentration, HVO: Hydrotreated vegetable oil at 13% intake O2 concentration, RME: Rapeseed methyl ester at 13% intake O2 concentration. CB: Carbon black Printex90. CB was not evaluated on day 90.  **Number of animals per group** Day 1: VEH=24, Extract=6, Particles=5-8. Day 28: VEH=18-20, Extract=6, Particles=3-6. Day 90: VEH=9-10, Extract=5-6, Particles=4-6. | | | | | | | |

**G. Neutrophil influx and estimated deposited metals on day 1 and surface area and EC on day 90**

Figure

**
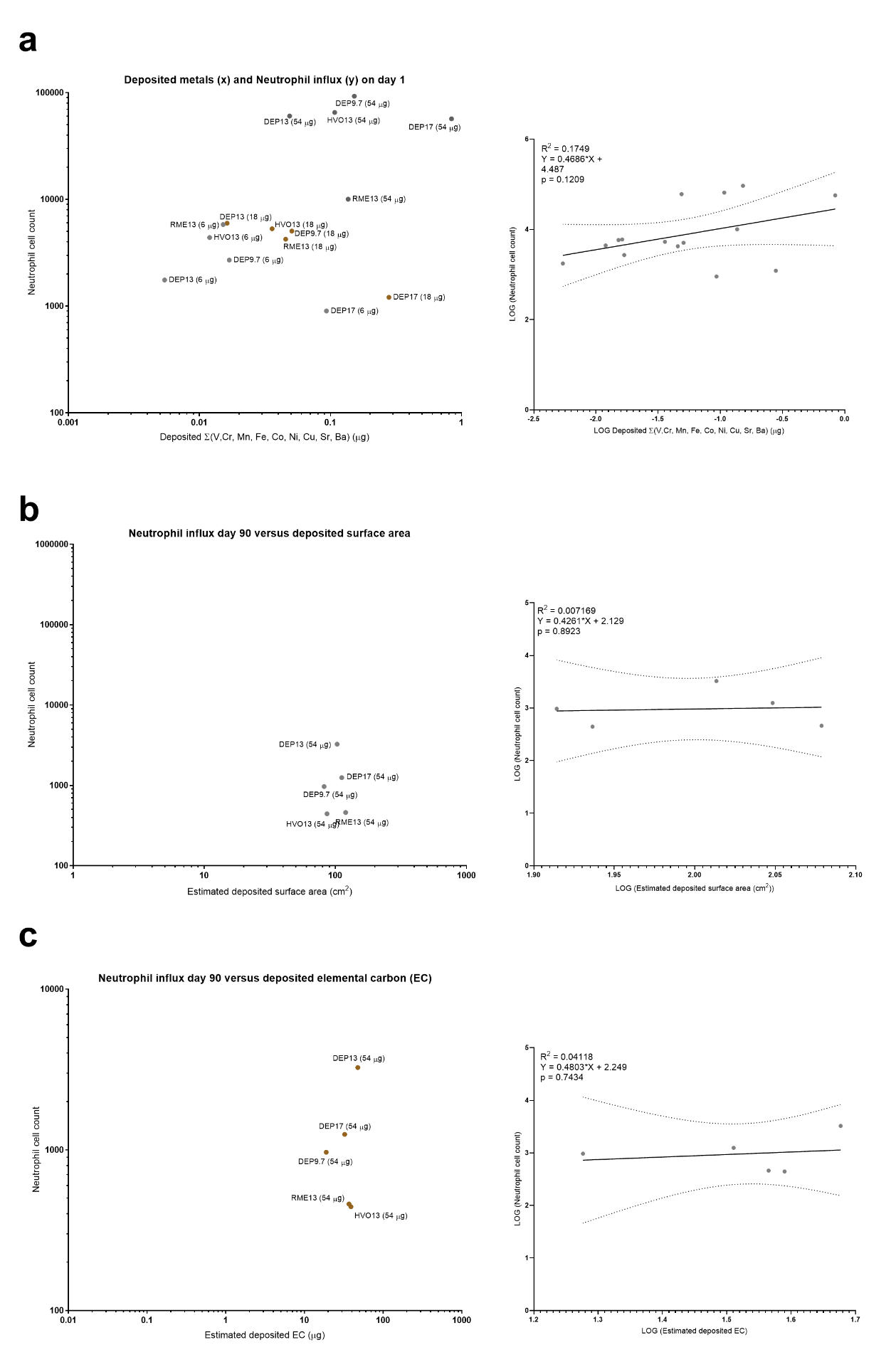
**

The estimated deposited specific surface area (SSA), elemental carbon (EC), organic carbon (OC) and PAHs were calculated by multiplying the physicochemical values by the dose (EC and OC: dose in µg * EC fraction = deposited EC in µg; for SSA: dose in g * SSA m2/g = deposited SSA in g; for PAH: dose in g * PAH µg/g = deposited PAH in µg). The data was log-transformed for the linear regression analysis.

**H. ROS formation and Tail Length in Comet assay on day 28**

Figure

**
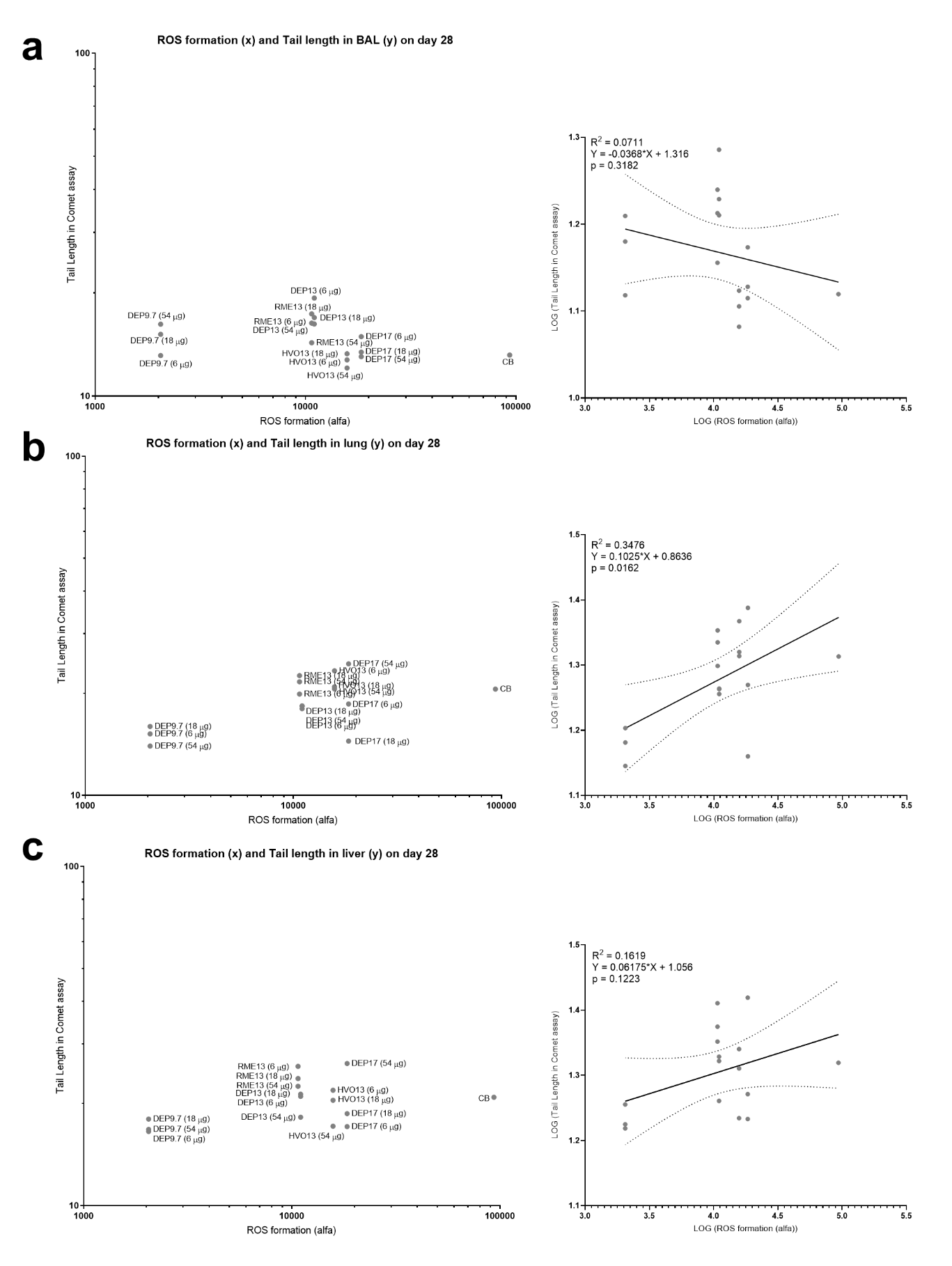
**

ROS formation correlations with Tail Length in Comet assay on day 28. a. BAL, original data (left panel), linear regression plot (right panel). b. Lung, original data (left panel), linear regression plot (right panel). c. Liver, original data (left panel), linear regression plot (right panel). The ROS is given in arbitrary alfa-values. The data was log-transformed for the linear regression analysis.

**I. Deposited PAH and Tail Length in Comet assay on day 28**

Figure

**
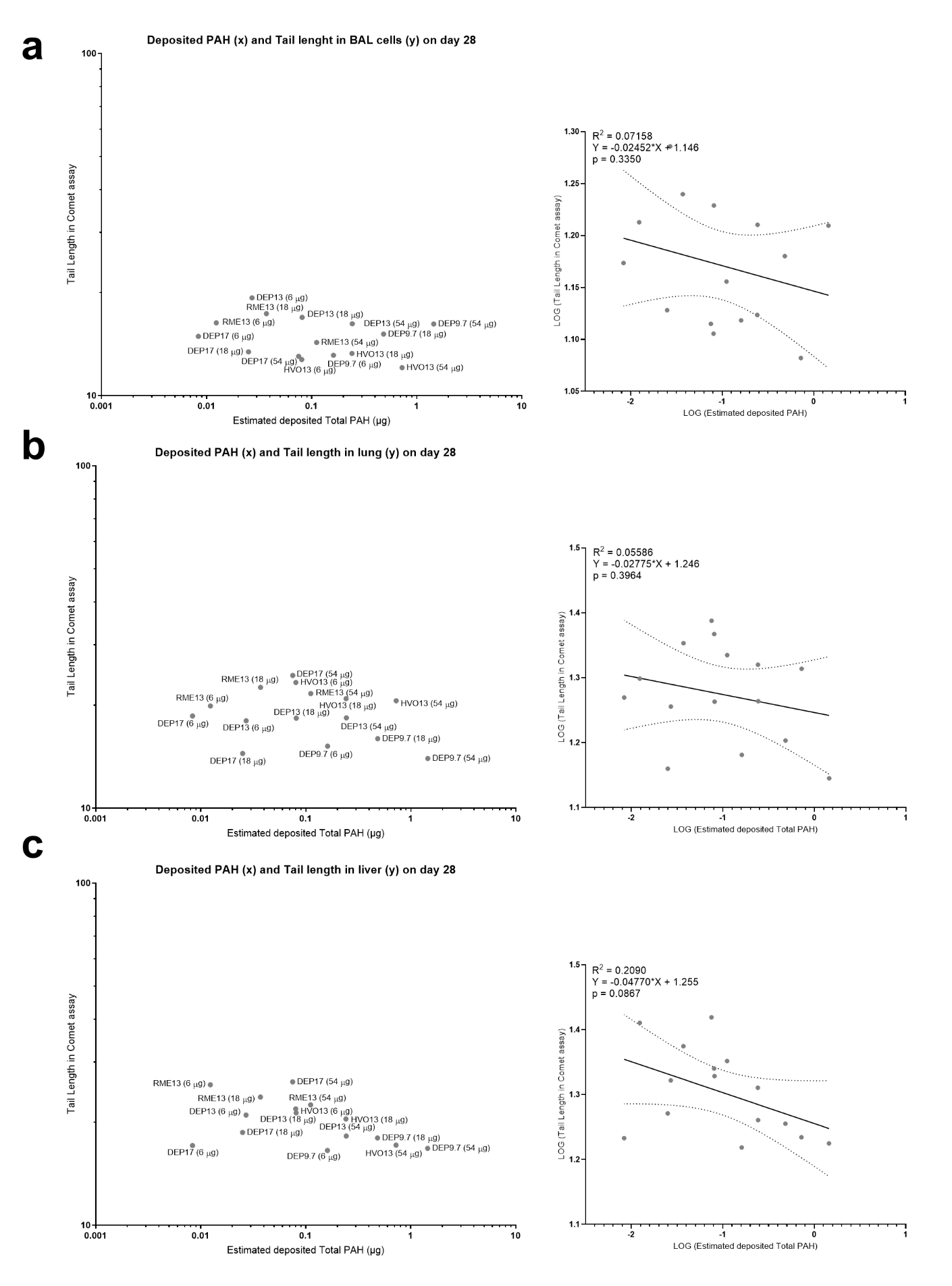
**

Estimated deposited Total (native) PAH correlations with Tail Length in Comet assay on day 28. a. BAL, original data (left panel), linear regression plot (right panel). b. Lung, original data (left panel), linear regression plot (right panel). c. Liver, original data (left panel), linear regression plot (right panel). The estimated deposited PAHs was calculated by multiplying the physicochemical values by the dose (for PAH: dose in g * PAH µg/g = deposited PAH in µg). The data was log-transformed for the linear regression analysis.

**J. Deposited PAH and Tail Length in Comet assay on day 90**

Figure

**
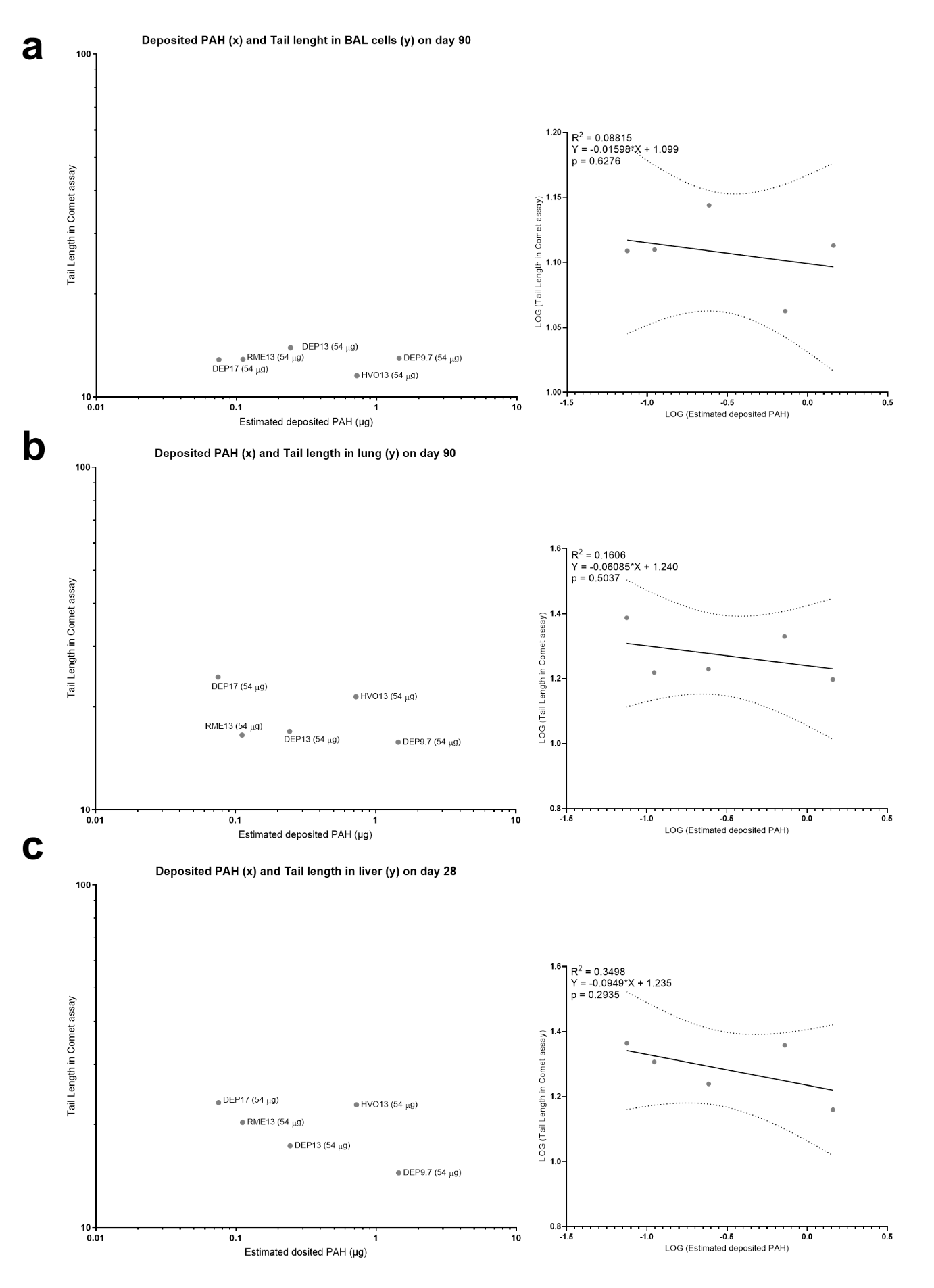
**

Estimated deposited Total (native) PAH correlations with Tail Length in Comet assay on day 90. a. BAL, original data (left panel), linear regression plot (right panel). b. Lung, original data (left panel), linear regression plot (right panel). c. Liver, original data (left panel), linear regression plot (right panel). The estimated deposited PAHs was calculated by multiplying the physicochemical values by the dose (for PAH: dose in g * PAH µg/g = deposited PAH in µg). The data was log-transformed for the linear regression analysis.

**K. Names, nominal masses, retention times and the associated deuterium labeled internal standards (IS) and recovery standards (RS) for PAHs**

Table

| **Table. PAHs and alkyl-PAHs** | | | | | |
| --- | --- | --- | --- | --- | --- |
| **Name** | **m/z** | **RT** | **IS** | **m/z** | **RT** |
| Naphthalene | 128 | 11.95 | D8 naphthalene | 136 | 11.90 |
| 2-methylnaphthalene | 142 | 13.61 | D8 acenaphthylene | 160 | 15.74 |
| 1-methylnaphthalene | 142 | 13.83 | D8 acenaphthylene | 160 | 15.74 |
| biphenyl | 154 | 14.77 | D8 acenaphthylene | 160 | 15.74 |
| 2.3-dimethylnaphthalene | 156 | 15.59 | D8 acenaphthylene | 160 | 15.74 |
| acenaphthylene | 152 | 15.74 | D8 acenaphthylene | 160 | 15.74 |
| acenaphthene | 154 | 16.18 | D10 acenaphthene | 164 | 16.10 |
| 2.3.5-trimethylnaphthalene | 170 | 17.27 | D10 acenaphthene | 164 | 16.10 |
| fluorene | 166 | 17.65 | D10 fluorene | 176 | 17.56 |
| 1-methylfluorene | 180 | 19.92 | D10 fluorene |  |  |
| phenanthrene | 178 | 21.88 | D10 phenanthrene | 188 | 21.75 |
| anthracene | 178 | 22.17 | D10 anthracene | 188 | 22.08 |
| 2-methylphenanthrene | 192 | 24.99 | D10 phenanthrene | 188 | 21.75 |
| 1-methylphenantrene | 192 | 25.74 | D10 phenanthrene | 188 | 21.75 |
| 1-methylanthracene | 192 | 25.82 | D10 phenanthrene | 188 | 21.75 |
| 3-methylphenanthrene | 192 | 25.83 | D10 phenanthrene | 188 | 21.75 |
| 2-phenylnaphthalene | 204 | 27.2 | D10 phenanthrene | 188 | 21.75 |
| fluoranthene | 202 | 29.73 | D10 fluoranthene | 212 | 29.61 |
| pyrene | 202 | 31.24 | D10 pyrene | 212 | 31.12 |
| 1-methylfluoranthene | 216 | 33.83 | D10 pyrene | 212 | 31.12 |
| retene | 234 | 34.06 | D10 pyrene | 212 | 31.12 |
| 1-methylpyrene | 216 | 35.5 | D10 pyrene | 212 | 31.12 |
| benzo(a)anthracene | 228 | 40.44 | D12 benzo(a)anthracene | 240 | 40.29 |
| chrysene | 228 | 40.68 | D12 chrysene | 240 | 40.49 |
| 2-methylchrysene | 228 | 40.68 | D12 chrysene | 240 | 40.49 |
| benzo(b)fluoranthene | 264 | 47.96 | D12 benzo(b)fluoranthene | 242 | 43.89 |
| benzo(k)fluoranthene | 264 | 48.19 | D12 benzo(k)fluoranthene | 252 | 48.12 |
| benzo(a)pyrene | 252 | 50.14 | D12 benzo(a)pyrene | 264 | 49.99 |
| perylene | 252 | 50.69 | D12 benzo(a)pyrene | 264 | 264 |
| indeno(1,2,3-c.d)pyrene | 276 | 56.86 | D12 indeno(1.2.3-c.d)pyrene | 288 | 57.03 |
| dibenzo(a,h)anthracene | 276 | 57.20 | D14 dibenzo(a.h)anthracene | 288 | 57.03 |
| benzo(g,h,i)perylene | 276 | 58.19 | D12 benzo(g.h.i)perylene | 288 | 58.05 |
|  |  |  | **RS**  Octachloror naphthalene (OCN) | 404 | 49.38 |

| **Table. Dibenzothiophenes (DBTs)** | | | | | |
| --- | --- | --- | --- | --- | --- |
| **Name** | **m/z** | **RT** | **IS** | **m/z** | **RT** |
| dibenzothiophene | 184 | 21.50 | D10 phenanthrene | 188 | 21.75 |
| 2-methyldibenzothiophene | 198 | 24.21 | D10 phenanthrene | 188 | 21.75 |
| 1-methyldibenzothiophene | 198 | 24.76 | D10 phenanthrene | 188 | 21.75 |
| 4-methyldibenzothiophene | 198 | 25.38 | D10 phenanthrene | 188 | 21.75 |
| 2,8-dimethyldibenzothiophene | 212 | 28.20 | D10 fluoranthene | 212 | 29.61 |
| 2,4,7-trimethyldibenzothiophene | 226 | 30.95 | D10 fluoranthene | 212 | 29.61 |
|  |  |  | **RS**  Octachlorornaphthalene (OCN) | 404 | 49.38 |

| **Table. Nitro-PAHs and oxy-PAHs** | | | | | |
| --- | --- | --- | --- | --- | --- |
| **Name** | **m/z** | **RT** | **IS** | **m/z** | **RT** |
| Nitro-PAHs | | | | | |
| 1-Nitro naphthalene | 173 | 17.82 | D9 2-Nitrofluorene | 220 | 30.80 |
| 2-Nitro naphthalene | 173 | 18.59 | D9 2-Nitrofluorene | 220 | 30.80 |
| 5-Nitro acenaphthalene | 199 | 27.87 | D9 2-Nitrofluorene | 220 | 30.80 |
| 2-Nitro fluorene | 211 | 30.81 | D9 2-Nitrofluorene | 220 | 30.80 |
| 9-Nitro anthracene | 223 | 31.49 | D9 2-Nitrofluorene | 220 | 30.80 |
| 9-Nitro phenanthrene | 223 | 33.65 | D9 2-Nitrofluorene | 220 | 30.80 |
| 3-Nitro fluoranthene | 247 | 42.62 | D9 3-Nitro fluoranthene | 256 | 42.70 |
| 4-Nitro pyrene | 247 | 42.98 | D9 6-Nitro chrysene | 284 | 50.70 |
| 1-Nitro pyrene | 247 | 43.99 | D9 6-Nitro chrysene | 284 | 50.70 |
| 2-Nitro pyrene | 247 | 44.54 | D9 6-Nitro chrysene | 284 | 50.70 |
| 7-Nitro benz(a)anthracene | 273 | 48.67 | D9 6-Nitro chrysene | 284 | 50.70 |
| 6-Nitro chrysene | 273 | 50.72 | D9 6-Nitro chrysene | 284 | 50.70 |
| 3-Nitro benzanthrone | 275 | 50.88 | D9 6-Nitro chrysene | 284 | 50.70 |
| 6-Nitro benzo(a)pyrene | 297 | 57.73 | D9 6-Nitro chrysene | 284 | 50.70 |
| Oxy-PAHs | | | | | |
| Naphthalene-1-aldehyde | 156 | 16.38 | D9 2-Nitrofluorene | 220 | 30.80 |
| 2-Naphthaldehyde | 156 | 16.42 | D9 2-Nitrofluorene | 220 | 30.80 |
| p-Fluorenone | 180 | 20.57 | D9 2-Nitrofluorene | 220 | 30.80 |
| Phenanthrene-9-aldehyde | 206 | 31.23 | D9 2-Nitrofluorene | 220 | 30.80 |
| 9.10-Anthraquinone | 208 | 26.92 | D9 2-Nitrofluorene | 220 | 30.80 |
| 1.4-anthraquinone | 208 | 29.01 | D9 2-Nitrofluorene | 220 | 30.80 |
| Benzanthrone | 230 | 41.34 | D9 3-Nitro fluoranthene | 256 | 42.70 |
| Benz(a)anthracene-7,12-dione | 258 | 43.73 | D9 3-Nitro fluoranthene | 256 | 42.70 |

**L. Calculation of benzo(a)pyrene equivalents (BaPeq)**

Table

| **Table. Calculated BaPeq for the 12 native PAHs with toxic equivalency factors (TEF) from Larsen and Larsen (1998)*** | | | | | | | |
| --- | --- | --- | --- | --- | --- | --- | --- |
|  | TEF | **DEP9.7** | **DEP13** | **DEP17** | **HVO13** | **RM13** | *Ref. NIST 2975* |
| phenanthrene | 0.0005 | 0.21 | 0.12 | 0.02 | 0.58 | 0.10 | 0.01 |
| anthracene | 0.0005 | 0.03 | 0.01 | 0.00 | 0.02 | 0.01 | 0.00 |
| fluoranthene | 0.05 | 83.85 | 7.07 | 8.11 | 79.57 | 16.26 | 1.17 |
| pyrene | 0.001 | 2.38 | 0.18 | 0.22 | 2.34 | 0.29 | 0.00 |
| benzo(a)anthracene | 0.005 | 6.89 | 0.03 | 0.08 | 0.94 | 0.03 | 0.00 |
| chrysene | 0.03 | 78.28 | 0.28 | 1.58 | 11.10 | 0.48 | 0.12 |
| benzo(b)fluoranthene | 0.1 | 415.81 | 20.76 | 4.07 | 54.84 | 2.78 | 0.82 |
| benzo(k)fluoranthene | 0.05 | 54.70 | 44.38 | 2.95 | 9.39 | 1.81 | 0.01 |
| benzo(a)pyrene | 1 | 3739.26 | 61.33 | 32.75 | 759.15 | 33.37 | 0.29 |
| indeno(1,2,3-c,d)pyrene | 0.1 | 207.21 | 0.23 | 0.87 | 70.25 | 0.21 | 0.09 |
| dibenzo(a,h)anthracene | 1.1 | 44.19 | 29.89 | 7.81 | 54.87 | 4.77 | 0.44 |
| benzo(g,h,i)perylene | 0.02 | 52.17 | 0.63 | 0.28 | 24.33 | 0.15 | 0.02 |
| **Sum BaPeq** |  | 4685 | 165 | 59 | 1067 | 60 | 3 |
| *Larsen, J.C., and P.B. Larsen: Air Pollution and Health. Cambridge, U.K.: Royal Society of Chemistry, 1998. | | | | | | | |
